# Naturally occurring substitution of an amino acid in a plant virus gene-silencing suppressor enhances viral adaptation to increasing thermal stress

**DOI:** 10.1371/journal.ppat.1011301

**Published:** 2023-04-03

**Authors:** Lina Cai, Mingqing Dang, Yawen Yang, Ruoxin Mei, Fan Li, Xiaorong Tao, Peter Palukaitis, Randy Beckett, W. Allen Miller, Stewart M. Gray, Yi Xu

**Affiliations:** 1 Department of Plant Pathology, Nanjing Agricultural University, Jiangsu Province, China; 2 State Key Laboratory for Conservation and Utilization of Bio-Resources in Yunnan, Yunnan Agricultural University, Kunming, China; 3 Department of Horticultural Sciences, Seoul Women’s University, Nowon-gu, Seoul, Republic of Korea; 4 Department of Plant Pathology, Entomology and Microbiology, Iowa State University, Ames, Iowa, United States of America; 5 Plant Pathology and Plant-Microbe Biology Section, School of Integrated Plant Science, Cornell University, Ithaca, New York, United States of America; 6 Emerging Pests and Pathogens Research Unit, USDA, ARS, Ithaca, New York, United States of America; Agriculture and Agri-Food Canada, CANADA

## Abstract

*Cereal yellow dwarf virus* (CYDV-RPV) encodes a P0 protein that functions as a viral suppressor of RNA silencing (VSR). The strength of silencing suppression is highly variable among CYDV-RPV isolates. In this study, comparison of the P0 sequences of CYDV-RPV isolates and mutational analysis identified a single C-terminal amino acid that influenced P0 RNA-silencing suppressor activity. A serine at position 247 was associated with strong suppressor activity, whereas a proline at position 247 was associated with weak suppressor activity. Amino acid changes at position 247 did not affect the interaction of P0 with SKP1 proteins from *Hordeum vulgare* (barley) or *Nicotiana benthamiana*. Subsequent studies found P0 proteins containing a P247 residue were less stable than the P0 proteins containing an S247 residue. Higher temperatures contributed to the lower stability and *in planta* and the P247 P0 proteins were subject to degradation via the autophagy-mediated pathway. A P247S amino acid residue substitution in P0 increased CYDV-RPV replication after expression in agroinfiltrated plant leaves and increased viral pathogenicity of P0 generated from the heterologous *Potato virus X* expression vector system. Moreover, an S247 CYDV-RPV could outcompete the P247 CYDV-RPV in a mixed infection in natural host at higher temperature. These traits contributed to increased transmission by aphid vectors and could play a significant role in virus competition in warming climates. Our findings underscore the capacity of a plant RNA virus to adapt to climate warming through minor genetic changes in gene-silencing suppressor, resulting in the potential for disease persistence and prevalence.

## Author summary

Climate change and extreme weather events pose an increased risk for crops to become infected with pathogens, which could severely affect global food security. However, the studies about the effects of climate change on plant virus diseases are still obscure. Yellow dwarf disease is one of the most important diseases of small grain cereals worldwide, which is caused by viruses belonging to the genera *Luteovirus* and *Polerovirus*. The gene encoding the P0 protein of poleroviruses has been reported to be the most rapidly evolving ORF of the *Polerovirus* genus. P0 functions as a viral suppressor of RNA silencing (VSR) and the strength of silencing suppression of P0 is highly variable among CYDV-RPV isolates. However, little is known about the molecular mechanism that determine P0 suppressor activity. Here, we identify a naturally occurring, single-amino-acid substitution in the CYDV-RPV P0 protein that enhances its protein stability to increasing thermal stress. This is the first study to identify a naturally occurring amino acid substitution in a plant virus-encoded protein that determines its adaption to increasing thermal stress. As climate change and extreme weather events occur frequently, we expect that our research can supply insight into how a plant RNA virus evolves to adapt climate change.

## Introduction

Yellow dwarf disease is one of the most important diseases of small grain cereals worldwide. It is caused by viruses belonging to the genera *Luteovirus* and *Polerovirus* in the family *Tombusviridae* and *Solemoviridae*, respectively [1]. *Cereal yellow dwarf virus*-RPV (CYDV-RPV) is a polerovirus member and causes economically important disease of small grains worldwide [2]. Yield losses vary year to year, ranging from 5 to 25%, depending on aphid vector populations and environmental conditions such as temperature, soil moisture, and soil fertility [3–6]. Extensive studies have shown that environmental factors play substantial roles in the yellow dwarf virus disease cycle. In general, high light intensity and lower temperatures of 15–18°C favor the expression of leaf discoloration symptoms in barley, oat and wheat plants, whereas, symptoms are mild at temperatures above 30°C [7]. CYDV-RPV is transmitted in a persistent, circulative manner by its main vector *Rhopalosiphum padi* (hence, the RPV suffix) and has increased in prevalence in recent years, in many countries [8,9].

Small RNA-mediated gene silencing plays evolutionarily conserved roles in gene regulation and defense against invasive nucleic acids in most eukaryotes, with specificity determined by small RNA molecules of 21–24 nucleotides (nt) in length [10,11]. To counter against the RNA silencing-mediated defense, plant and some animal viruses overcome host antiviral silencing by encoding diverse viral suppressors of RNA silencing (VSRs) [12]. The P0 protein encoded by the 5′-proximal open reading frame (ORF) of poleroviruses has been shown to suppress RNA silencing. The suppressor activities of polerovirus P0 proteins have been extensively studied and they are thought to target AGO1 by means of their F-box-like motifs interacting with E3-ligase S-phase kinase-related protein-1 (SKP1) [13]. The subsequent degradation of AGO1 is via the autophagy pathway [14]. However, the P0 from the IM isolate of *Potato leafroll virus* (PLRV) does not interact with SKP1 yet still mediates AGO1 degradation [15], suggesting there are multiple mechanisms by which P0 proteins can mediate silencing suppression. P0 proteins from two viruses, CYDV-RPV-NY and CYDV-RPS, both interacted with Arabidopsis E3-ubiquitin ligase protein ASK2 to destabilize AGO1 [16]. Independent of the F-box domain, aromatic amino acids in the C-terminus of P0 from PLRV and *Melon aphid-borne yellows virus* were shown to play a role in suppression of RNA silencing [15,17]. Interestingly, a recent study found *Brassica yellows virus* (BYV) P0 protein could be degraded through the proteasome and autophagy pathways, and the interaction between P0 and plant SKP1 facilitates stability of P0 in vivo [18].

In nature, the strength of silencing suppression activity is highly variable among isolates of polerovirus species [16,19]. The P0 proteins of most *Beet mild yellowing virus* isolates exhibit measurable RNA silencing-suppressor activities whereas those from *Beet mild yellowing virus* isolates N32 and 26, and several related beet chlorosis virus isolates from Europe lack suppressor functions [20]. The P0 of a European isolate of PLRV (PLRV-Dutch) is a weak suppressor of local RNA silencing, whereas the P0 of PLRV isolates from Australia and Inner Mongolia have strong locally acting suppressor activity and systemic silencing suppressor activity [15,19]. P0 from an infectious clone of CYDV-RPV (P0-NY), derived from an isolate collected in New York in 1957 showed no suppression activity when tested on assays based on *Beet necrotic yellow vein virus* multiplication, complementation and *Agrobacterium tumefaciens*-mediated expression assays [21,22]. In contrast, the P0 from the CYDV-RPV 005 isolate (GenBank No. EF521827.1, P0-005), collected in Missouri in 2006, showed a very strong suppressor activity using the same *Agrobacterium*-mediated transient expression system [19]. The strength of silencing suppression activity of P0 has been shown to determine disease severity. For example, CYDV-RPS causes corkscrew leaf curling and notching symptoms in wheat and oat, in addition to yellowing and dwarfing caused by the milder virus CYDV-RPV [16]. Correlated with this extreme disease severity, the P0 protein of CYDV-RPS produces stronger and more durable suppression of silencing in *Nicotiana benthamiana* [16]. Thus, P0 may be responsible for the more severe symptoms of CYDV-RPS.

The effects of global climate change and extreme weather on infectious diseases mainly focus on mammalian viruses. Carlson *et al*., showed climate change could drive more than 15,000 new cases of mammals transmitting viruses to other mammals [23]. However, the studies about the effects of climate change on plant virus diseases are still obscure. In this study, we investigated the correlation of P0 suppressor activities with virulence and P0 sequences among CYDV-RPV isolates. We demonstrated that a single C-terminal amino acid in the CYDV-RPV P0 determined P0 protein stability and influenced P0 suppressor activity to increasing thermal stress. The strong suppressor improved viral fitness to increasing thermal stress, suggesting that P0 protein stability plays a significant role in the persistence and prevalence of CYDV-RPV isolates as the climate changes.

## Results

### P0s from two different CYDV-RPV isolates display different VSR activities

**Fig 1:**
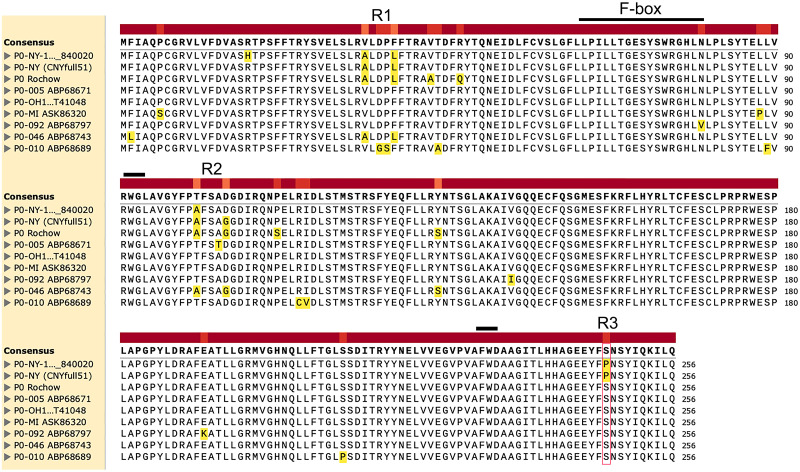
Amino acid sequence alignment of all CYDV-RPV P0 available. The six differences in sequence between P0-NY and P0-005 are indicated by the regions R1-R3. The red box indicates the position of the amino acid 247. The F-box domain, WG, FW and the different domain R1, R2, R3 are outlined or underlined in black.

Previous studies have shown that CYDV-RPV P0s from different isolates have varying VSR activities [16,21,22]. To further investigate the differences in CYDV-RPV P0 VSR activities and identify the motifs responsible for the suppression differences, we compared sequences of different CYDV-RPV-NY genomes. CYDV-RPV-NY was first isolated by Bill Rochow in 1957. A descendent of this isolate, including ORF 0 (GenBank No. NP_840020), was mostly sequenced in 1991 at Purdue University [24]. Also descended from this sequence, was a full-length infectious clone (pCNYfull51, which represents just one genomic sequence in the infecting quasispecies) obtained in 2002 at Iowa State University and is used in this paper. We refer to P0 from this clone as P0-NY. In 2017, a third sequence of this isolate was obtained at Cornell University after 60 years of passaging by aphid transmission in Coast Black oats. We call the P0 encoded in this sequence P0-Rochow. In P0, the three CYDV-RPV-NY sequences vary at six amino acid positions (Fig 1). Because of sequence variation within a virus quasispecies, different culture conditions, as well as many serial passages via aphid transmission in different labs, this sequence variation is unsurprising. Upon comparison with the other CYDV-RPV genomes in GenBank, we found that P0 from the original 1991 sequence (NP_840020) and in our infectious clone (P0-NY) contain a proline at position 247, while the others, including P0-Rochow harbor a serine at this position. To determine the importance of this difference, here we compare the biological activity of the cloned P0-NY, with that of CYDV-RPV genome 005 (P0-005, ABP68671) which has been reported as a strong suppressor [19]. There are six amino acid differences between P0-NY and P0-005, located in three regions designated R1-R3 (Fig 1); the P0-Rochow contained additional amino acid sequence differences, but similar to P0-005, it contained a serine at position 247 in the R3 (Fig 1). The F-box like motif, GW/WG motif, and the C-terminal aromatic amino acids previously reported to be essential for polerovirus P0 gene silencing suppressor activity [13,15,17,25] were conserved among all P0s, except for an N80V substitution at the last position of the F-box-like motif in two isolates (Fig 1).

**Fig 2:**
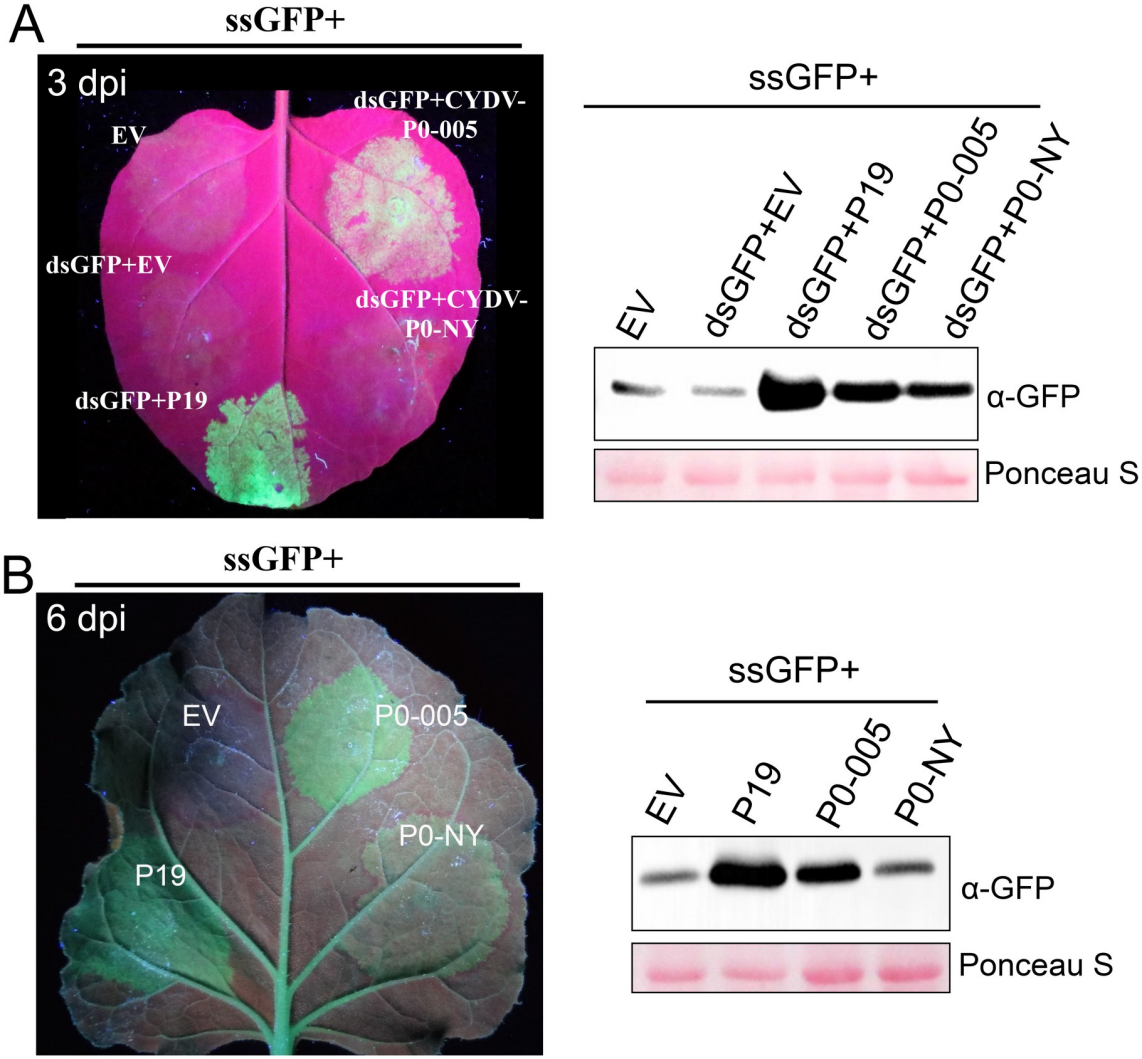
Silencing suppression activity of P0-NY and P0-005. (A) Leaves of *N*. *benthamiana* plants were co-agroinfiltrated with bacteria harboring a vector encoding GFP-targeting dsRNA (dsGFP), a vector encoding sense GFP (ssGFP) and either an empty vector (EV), or a vector encoding CYDV-P0-NY, CYDV-P0-005, or P19 (as a positive control). The leaves were photographed at 3 dpi under a hand-held long-wavelength UV lamp. (B) Leaves of the *N*. *benthamiana* 16c plants were agroinfiltrated with bacteria harboring a vector encoding GFP plus either EV or a vector encoding CYDV-P0-NY, CYDV-P0-005, or P19. The leaves were photographed at 6 dpi under a hand-held long-wavelength UV lamp. GFP accumulation was also confirmed by western blot analysis and shown right the leaf photographs.

Suppressor activities of CYDV-RPV P0-NY and P0-005 were determined by co-expressing the candidate genes with a sense green fluorescent protein gene (sGFP) plus the inverted repeat GFP gene in *N*. *benthamiana* leaves. P0-005 showed strong suppressor activity, whereas P0-NY showed weak suppressor activity at 3-days post-infiltration (dpi) (Fig 2A). Similar results were obtained when the P0 genes were co-expressed with an sGFP in the *N*. *benthamiana* line 16c, which already has an integrated GFP transgene [26] (Fig 2B). At 6 dpi, no GFP fluorescence was observed when sGFP was co-expressed with P0-NY, whereas GFP fluorescence was still intense when co-expressed with P0-005 (Fig 2B). Western blot analyses confirmed that GFP protein accumulation was consistent with GFP fluorescence levels (Fig 2). Consistent with other reports [19,27], CYDV-RPV P0s did not block the short-range silencing signal to neighboring cells manifested as a red halo surrounding the infiltrated areas (Fig 2B).

### Serine at position 247 is important for CYDV-RPV P0 VSR activity

**Fig 3:**
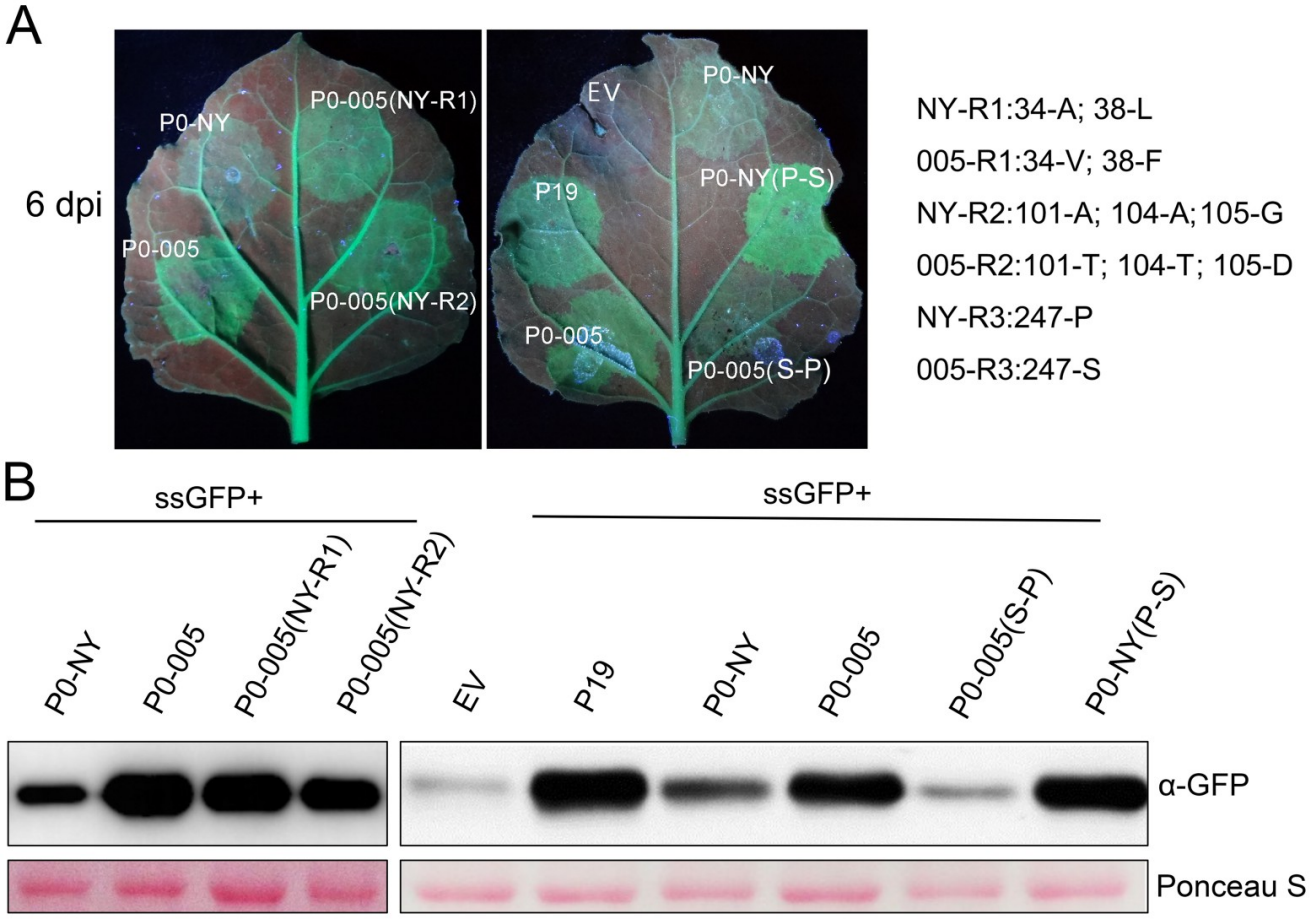
Silencing suppression activity of P0-NY, P0-005 and substitution mutants. (A) Leaves of the *N*. *benthamiana* 16c plants were co-agroinfiltrated with bacteria harboring a GFP-expressing vector (GFP) and either a vector expressing P19 (as a positive control) or a vector expressing CYDV-P0 variant mutants. Empty vectors are denoted as “EV”. The leaves were photographed at 6 dpi under a hand-held long-wavelength UV lamp. The sequence differences in regions 1–3 (R1-R3) between P0-NY and P0-005 are shown. (B) GFP accumulation was also confirmed by western blot analysis.

Mutational analysis of P0-005 and P0-NY was conducted to identify which of the six amino acids that distinguish the two proteins were responsible for differences in the silencing suppression phenotype. Introducing the P0-NY-specific residues into P0-005 in the R1 and R2 motifs (Fig 1) had no effects on the suppressor activity, whereas the S247P substitution in P0-005 decreased the suppressor activity, and the reciprocal P247S substitution in P0-NY (mutant R3) increased the suppressor activity (Fig 3). Taken together these results demonstrate that amino acid 247 is responsible for the observed differences in the P0-NY and P0-005 phenotypes. A comparison of all available online CYDV-RPV P0 sequences revealed that two CYDV isolates collected in the U.S. in New York State 30 years ago contained a proline at loci 247 of P0 (NP_840020.1; AAA42867.1); all other seven isolates have a serine at this position. It is noteworthy that all these seven isolates with serine at loci 247 of P0 were collected after the year of 2007 (S1 Fig).

### CYDV-RPV P0 with a serine at position 247 shows stronger protein stability

**Fig 4:**
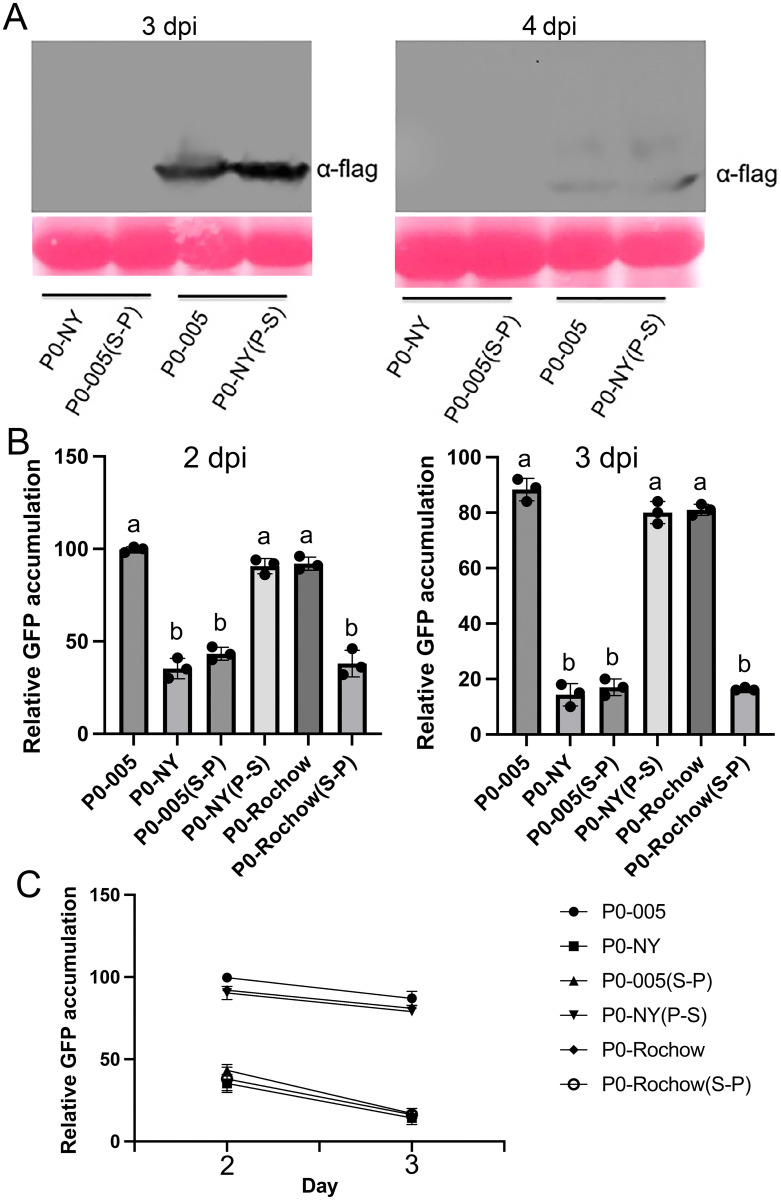
Differences in P0 protein accumulation in leaves transiently expressing P0-NY, P0-005 and P0 amino acid 247 substitution mutants. (A) Leaves of the *N*. *benthamiana* plants were agroinfiltrated with bacteria containing pBlin-P0-NY-flag, pBlin-P0-005(S-P)-flag, pBlin-P0-005-flag or pBlin-P0-NY(P-S)-flag. Protein from each of the infiltrated tissues was extracted at 3 dpi and 4 dpi and then P0-flag accumulation was confirmed by western blot analysis. (B) Leaves of the *N*. *benthamiana* plants were agroinfiltrated with bacteria containing pBlin-GFP-P0-NY, pBlin-GFP-P0-005(S-P), pBlin-GFP-P0-005, pBlin-GFP-P0-NY(P-S), pBlin-GFP-P0-Rochow or pBlin-GFP-P0-Rochow(S-P). The GFP-P0 accumulation was quantified by western blot at 2 dpi and 3 dpi. The relative protein level of P0-005 at 2 dpi was normalized to rubisco, and this value was set as standard 100. Three replicates were performed by western blot. (C) The graph was generated based on the value of P0 accumulation value from (B). Letters (a, b) above the bars in (B) indicate significant differences revealed by Dunn’s multiple comparisons test p<0.05.

Western blots were used to analyze the protein accumulation of P0-NY, P0-005, P0-NY(P-S) and P0-005(S-P) in *N*. *benthamiana* leaves. P0-005 and P0-NY(P-S) accumulated to higher levels than did P0-NY and P0-005(S-P) (Fig 4A). P0-NY and P0-005(S-P) were barely detectable, either at 3 dpi or 4 dpi (Fig 4A). To rule out the possibility that the transcription of P0(s) was different, cycloheximide (CHX) utilized, and the result revealed a degradation pattern similar to that observed without CHX, as shown in S2 Fig. To further investigate the difference in P0 protein accumulation, genes encoding wild-type P0-NY, P0-005 and the mutants P0-NY(P-S) and P0-005(S-P) were inserted into the binary vector fused to the 3’-terminus of the GFP ORF (pBlin-GFP-P0). Following agroinoculation into *N*. *benthamiana* leaves, the GFP-P0 protein expression level was monitored after 2 and 3 dpi using confocal microscopy and western blot. Fluorescence accumulation in leaves infiltrated with Agrobacterium harboring plasmids expressing P0-005 and P0-NY(P-S) fused to GFP was significantly higher than in leaves infiltrated with Agrobacterium harboring plasmids expressing P0-NY and P0-005(S-P) fused to GFP (S3A Fig). More inclusion bodies near the perinuclear space were observed when a proline was at position 247 (S3A and S3B Fig). The relative levels of fluorescence of the GFP-P0 fusion proteins in leaves were verified by western blot (Fig 4B) and P0 with a proline at 247 loci degraded faster than that with a serine (Fig 4C). These results suggest that the reduced silencing suppression activity of the P0-NY is due to an inability to accumulate *in planta*, rather than to a loss of silencing suppression activity *per se*.

The P0-Rochow protein contained additional amino acid sequence differences from either P0-NY and P0-005, although like P0-005, P0-Rochow contained a serine at position 247 (Fig 1). To test whether this amino acid could also determine its protein stability, genes encoding P0-Rochow and P0-Rochow(S-P) were fused to the sequences at the 3’-terminus of the GFP ORF in pBlin-GFP-P0 and after infiltration of leaves with bacteria harboring these plasmids, the GFP accumulation levels were detected by western blot and confocal microscopy observation at 2 dpi and 3 dpi. There was a significant reduction in GFP accumulation driven by P0-Rochow (S-P) compared to the wild-type P0-Rochow (Figs 4B and S3). Taken together these data led us to hypothesize that the change of amino acid 247 from serine to proline in CYDV-RPV P0 may affect the protein structure, which we examined *in silico*. AlphaFold2 algorithm [28] was used to predicted the P0-NY 3D structure and the STRUM server, a structure-based method for predicting the fold stability change (ΔΔG) of protein molecules upon single-point SNP mutations [29], predicts that the P0 protein stability would decrease if serine 247 was changed to a proline (S4A Fig).

### Proline at position 247 contributes to the lower stability of P0

**Fig 5:**
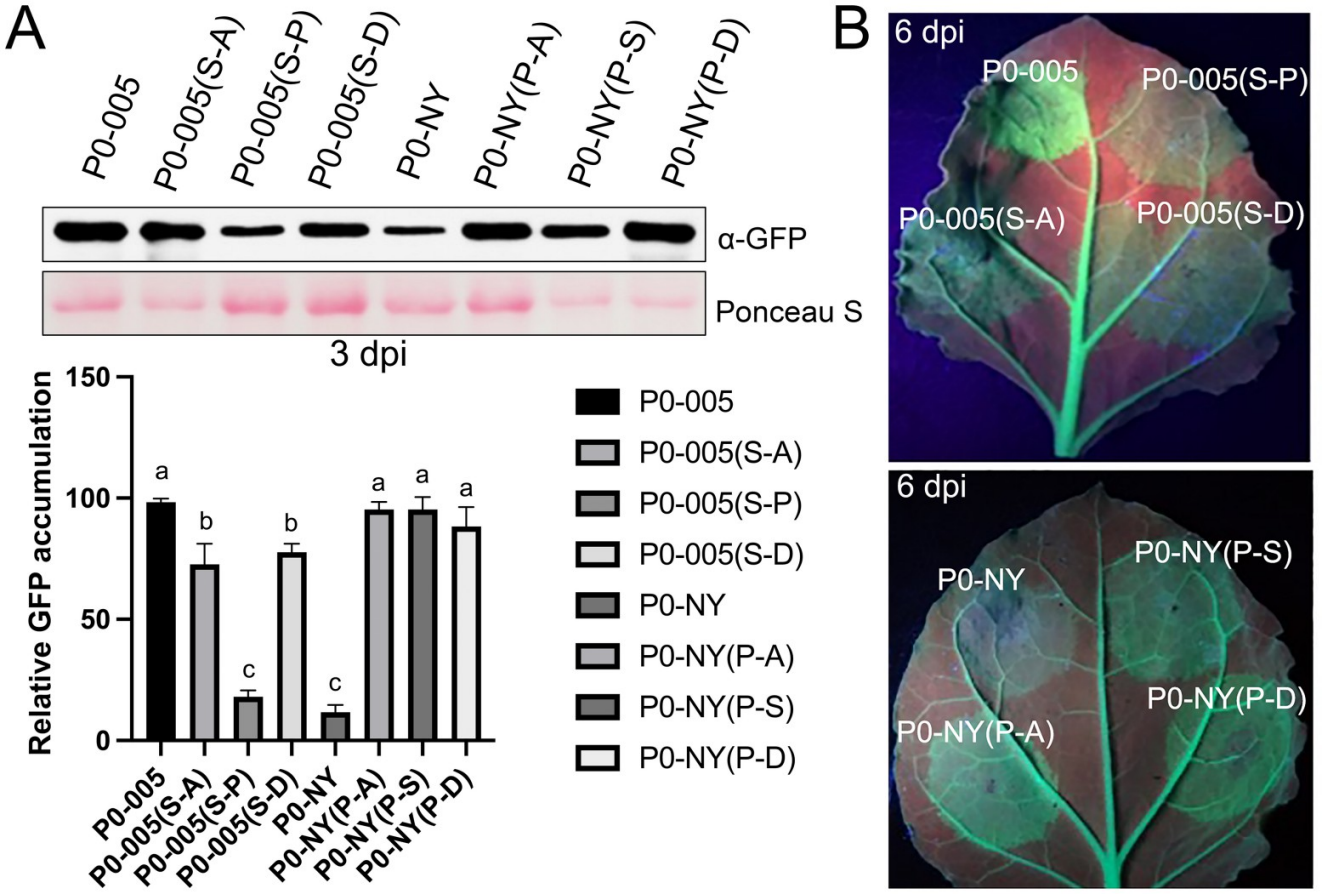
The P0 protein accumulation and gene-silencing suppressor activity of P0-NY, P0-005 and phosphorylation mimic mutants. (A) Leaves of the *N*. *benthamiana* plants were agroinfiltrated with bacteria containing pBlin-GFP-P0-005, pBlin-GFP-P0-005 (S-A), pBlin-GFP-P0-005 (S-P), pBlin-GFP-P0-005 (S-D), pBlin-GFP-P0-NY, pBlin-GFP-P0-NY(P-A), pBlin-GFP-P0-NY(P-S) or pBlin-GFP-P0-NY (P-D). The GFP-P0 accumulation was monitored by western blot at 3 dpi (Top panel). Bottom panel: the relative protein level of GFP-P0-005 was normalized to rubisco, and this value was set as standard 100. Letters (a, b, c) above the bars indicate significant differences revealed by Dunn’s multiple comparisons test p<0.05. Three replicates were performed by western blot. (B) Leaves of the *N*. *benthamiana* 16c plants were co-agroinfiltrated with a GFP-expressing vector (GFP) and either pBlin-P0 or one of its variants or mutants. The leaves were photographed at 6 dpi under a hand-held long-wavelength UV lamp.

Proline is the sole amino acid incapable of forming main-chain hydrogen bonds. Also, sequence analysis plus prediction software [30] identified S247 as a likely position for phosphorylation (S4B Fig). To investigate the potential importance of this chemical feature of proline and phosphorylation capability of serine, additional P0-NY and P0-005 mutants were constructed in which P247 and S247 was changed to either alanine, P0-NY(P-A) and P0-005(S-A), or aspartic acid, P0-NY(P-D) and P0-005(S-D). Alanine cannot be modified by phosphorylation and aspartic acid is chemically similar to phospho-serine [31]. Following agroinfiltration of bacteria harboring plasmids expressing GFP gene fusions with these modified P0 genes into *N*. *benthamiana*, the infiltrated areas of the leaves were examined by western blot at 3 dpi. As presented, mutant P0-NY(P-A) and P0-NY(P-D) showed a higher level of GFP accumulation compared to the wild-type P0-NY (Fig 5A). By contrast, there was a slight decrease in GFP expression of P0-005(S-A) and P0-005(S-D) compared to the wild-type P0-005 (Fig 5A). In addition, there was a slight decrease in GFP accumulation of P0-005(S-A) and P0-005(S-D) compared to the wild-type P0-005 (Fig 5A). These results suggest the stability/activity of P0 was not caused by serine phosphorylation of amino acid 247. Furthermore, an increase of P0-NY(P-A) and P0-NY(P-D) accumulation was linked to stronger P0 suppressor activity which was indicated by photograph taken under UV light at 6 dpi (Fig 5B).

### CYDV-RPV P0 is degraded through the autophagy pathway

**Fig 6:**
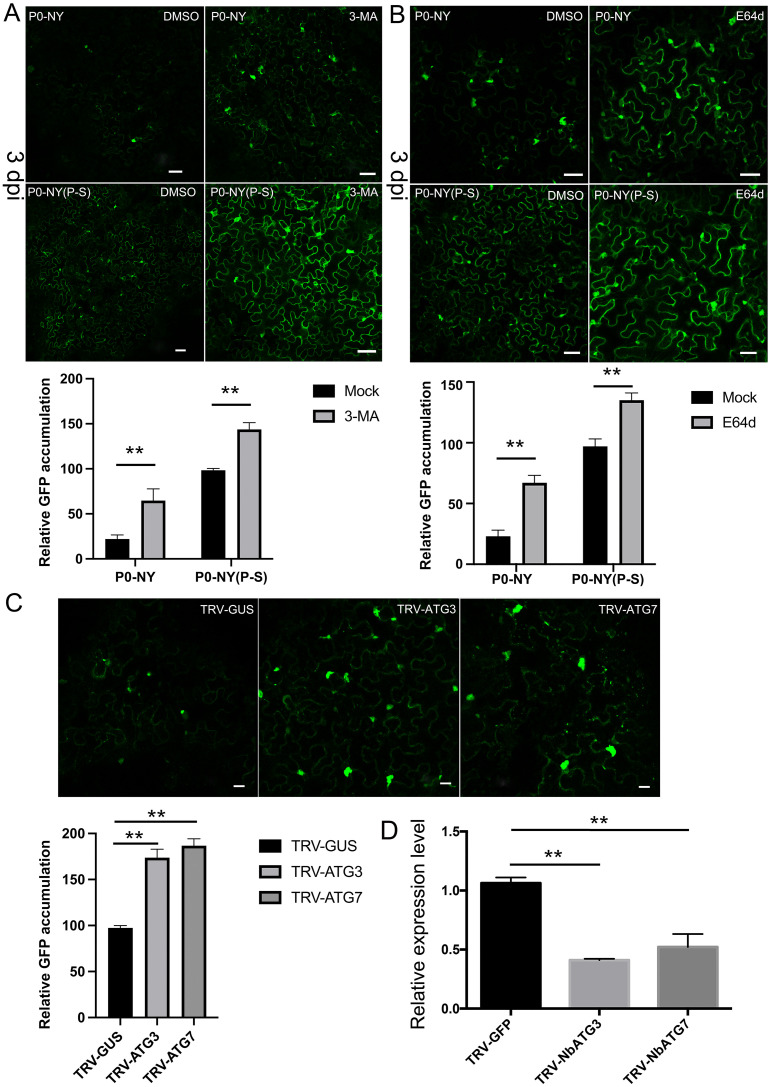
P0-NY is degraded through the autophagy pathway. In vivo degradation inhibition analysis of P0-NY and P0-NY(P-S) accumulation after adding autophagy pathway inhibitors 3-MA (A), or E64d (B). *A*. *tumefaciens* containing pBlin-GFP-P0-NY and pBlin-GFP-P0-NY(P-S) constructs were infiltrated for transient expression side by side on either halves of independent *N*. *benthamiana* leaves, 16 h prior to inhibitor treatment. DMSO infiltration was used as a control. The GFP-P0 accumulation was monitored by western blot at 3 dpi. Bars: 50 μm. The protein accumulation level was also quantified by western blot in the lower panel, with three replicates employed to ensure the reliability of the results. The relative protein level of P0-NY(P-S) with DMSO infiltration was normalized to rubisco, and this value was set as standard 100. **p < 0.01 by t-test analysis. (C) Effect of *NbATG3*, *NbATG7* knockdown on the protein stabilization of P0-NY in *N*. *benthamiana*. A TRV-based virus induced gene silencing system was used to silence NbATG3, NbATG7, respectively. A TRV vector carrying a GUS fragment was used as a control. At 3 wpi, the silenced upper leaves were infiltrated with *Agrobacterium* harboring a construct containing pBlin-GFP-P0-NY. Accumulation of GFP-P0 proteins was detected by confocal microscopy and western blot at 3 dpi, respectively. **p < 0.01 by t-test analysis. Bars: 20 μm. (D) NbATG3 or NbATG7 mRNA was analyzed by quantitative, reverse-transcription PCR, respectively. *N*. *benthamiana* actin was used as an internal control. **p < 0.01 by t-test analysis.

P0-NY was consistently observed to be concentrated in inclusion bodies near the perinuclear space, similar to aggresomes (S3 Fig and S1 Movie). Protein aggregates are processed by autophagy via sequestration in autophagosomes and delivery to lysosomes for clearance [32]. To investigate the potential involvement of the lysosomal autophagy pathway in the degradation of CYDV-RPV P0, the compounds 3-methyl adenine (3-MA) and E64d were used to inhibit the autophagy pathway [33,34]. Both GFP-P0-NY and GFP-P0-NY(P-S) accumulated to higher levels in leaves treated with 3-MA (Figs 6A, 6B and S5A), relative to their levels in either untreated leaves or leaves treated with DMSO, suggesting that both P0-NY and P0-NY(P-S) are degraded through the autophagy pathway. E64d treatment showed more protein aggregates, indicating that the degradation of GFP-P0 inside autolysosomes was inhibited (Figs 6B and S5A). Notably, P0-NY(P-S) seems to be more resistant to degradation than P0-NY (Figs 6A, 6B and S5A). To further test the conclusion that P0-NY was degraded through the autophagy pathway, sequences (200 nt) corresponding to the ATG3 and ATG7 genes in the autophagy pathway were cloned into the *Tobacco rattle virus* (TRV)-based virus induced gene silencing vector to induce silencing of autophagy machinery, respectively. After confirming the silencing efficiency at 3 weeks post inoculation (wpi) (Fig 6D), GFP-P0 protein accumulation was analyzed by western blot. The level of P0-NY in autophagy-machinery-silenced plants was higher than that in the non-silenced control plants (Figs 6C and S5B), further supporting the conclusion that the autophagy machinery is responsible for P0 degradation.

### P0 S247P substitution has no effect on P0 interacting with NbSKP1 and HvSKP1 at 22°C

**Fig 7:**
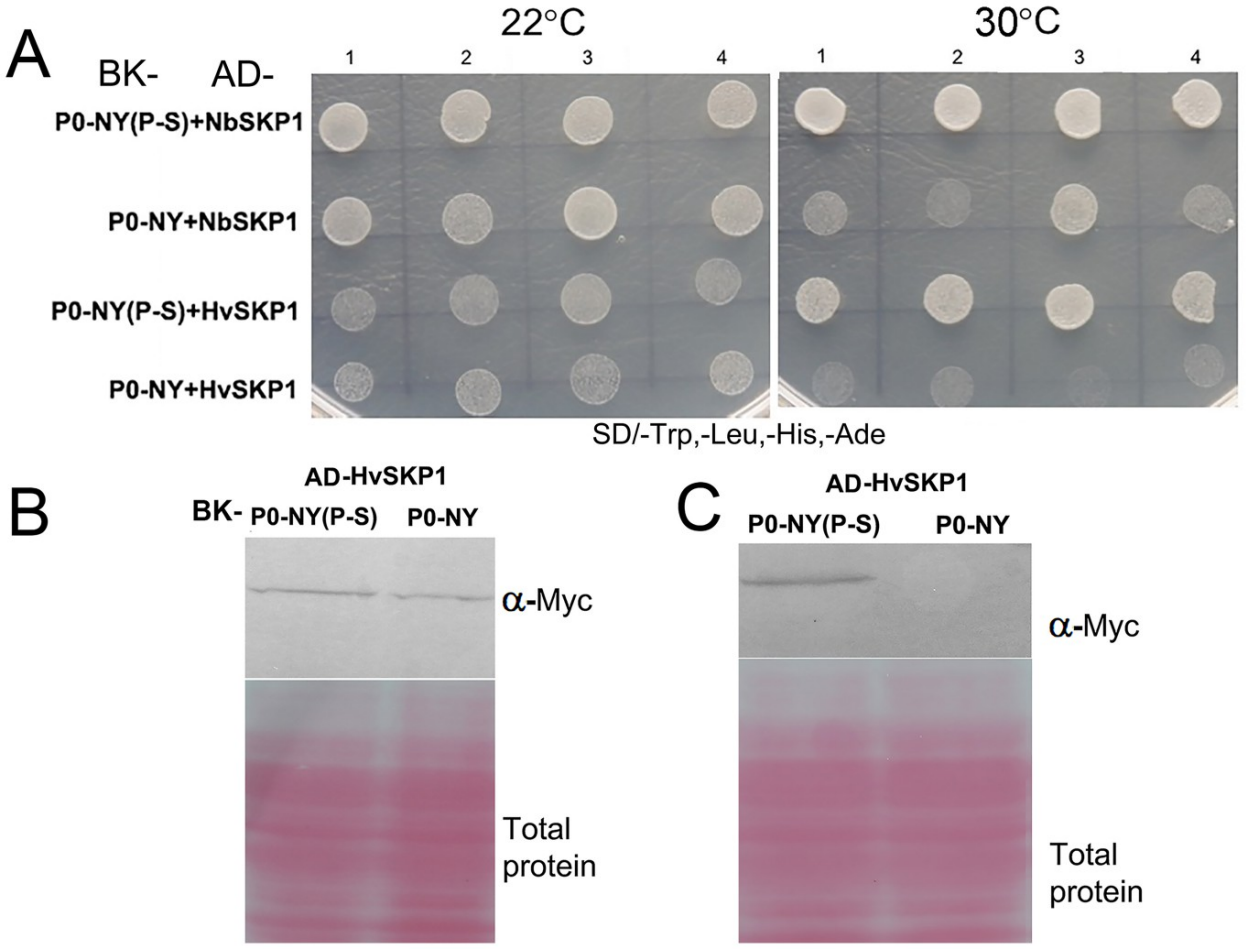
Detection of interaction between P0-NY or P0-NY(P-S) with NbSKP1 or HvSKP1 at 22°C and 30°C in the yeast two-hybrid system. P0-NY and P0-NY(P-S) were cloned into the bait vector pGBKT7, while NbSKP1 and HvSKP1 were cloned separately into the prey vector pGADT7. The bait and prey vectors were co-transferred into the yeast AH109 strain. Successful yeast mating resulted in vigorously growth on synthetic dropout (SD) media lacking Trp and Leu (SD/-WL). Interaction was indicated by yeast growth on SD media lacking Ade, Trp, Leu, and His. Four independent colonies were used as replicates. (B) and (C) Yeast strain AH109 co-transformed with pGBKT7-P0-NY/P0-NY(P-S) and pGADT7-HvSKP1 was spotted onto synthetic dextrose dropout medium, SD/-Trp/-Leu. After selecting the positive clone by PCR, the yeast was incubated in liquid medium SD/-Trp/-Leu at 22°C (B) or 30°C (C) overnight. Yeast total proteins were extracted, and western blot analysis was performed using Myc-tag antibody to detect the P0 protein accumulation. Total protein was used as a control.

Many polerovirus P0 proteins are known to interact via the F-box domain with the host plant E3-ubiquitin ligase protein SKP1 to mediate AGO1 protein degradation [16,25]. Furthermore, Li *et al*., (2019) reported that the interaction between BYV P0 and SKP1 facilitated the stability of P0 *in vivo* [18]. To determine if amino acid 247 could also affect the interaction between CYDV-P0 and SKP1, sequences encoding CYDV-P0-NY (P247S) and wild-type CYDV P0-NY were inserted into the pGBKT7 bait vector (Takara Bio, USA). Four independent colonies co-transformed with bait (pGBKT-P0) and prey (pGADT-SKP1) plasmids were streaked on media without tryptophan, leucine, histidine, and adenine (SD/-Trp,-Leu,-His,-Ade). This yeast two-hybrid interaction results indicated that both P0s interacted with SKP1 from either *N*. *benthamiana* or the natural host barley (*Hordeum vulgare* L.) (Fig 7A). While this interaction was expected, due to the conservation of the F-box domain in P0-NY and P0-005. Interestingly, we found there was a temperature effect on the interaction between P0 and SKP1. At 22°C there was a strong interaction between either P0-NY or P0-NY(P-S) and NbSKP1/HvSKP1, but the interaction was reduced with P0-NY when the temperature was raised to 30°C (Fig 7A). To determine if the temperature effect was related to P0-NY instability, yeast strain AH109 co-transformed with pGBKT7-P0-NY/P0-NY(P-S) and pGADT7-HvSKP1 was spotted onto synthetic dextrose dropout medium, SD/-Trp/-Leu. After selecting positive clones using PCR, the yeast were incubated in liquid medium SD/-Trp/-Leu at 30°C overnight and protein extracts were tested in western blots. The protein accumulation levels of wild-type P0-NY and P0-NY (P-S) were similar at 22°C but P0-NY was barely detectable at 30°C (Fig 7B and 7C) indicating the weak silencing suppressor, P0-NY, was not stable at higher temperatures in yeast, whereas the strong silencing suppressor, P0-NY(P-S) was stable at both temperatures. Moreover, the interactions of P0-NY and P0-NY (P-S) with NbSKP1 and HvSKP1 were also confirmed by using bimolecular fluorescence complementation (BiFC) assay, as shown in S6 Fig. Notably, the weaker fluorescence signal observed in the interaction between P0-NY and NbSKP1/HvSKP1 provides further evidence that P0-NY is less stable than P0-NY(P-S) under the 26°C/24°C day/night conditions.

### P0 is unstable under thermal stress conditions *in planta*

The P0-NY instability at higher temperature in yeast and *N*. *benthamiana* leaves led us to examine protein stability at various temperatures *in planta*. *Agrobacterium* carrying pBin-GFP-P0-NY or pBin-GFP-P0-NY(P-S) was infiltrated on opposite halves of *N*. *benthamiana* plants, and the plants were kept in growth chambers maintained at 20°C, 24°C, or 28°C with all other conditions held constant. GFP expression was measured at 72 hpi by confocal microscopy and western blot analysis. P0-NY expression was only slightly less than P0-NY(P-S) at 20°C but decreased dramatically at 24°C and 28°C (S7A and S7B Fig). Our previous VSR assays, which indicated P0-NY was a weak silencing suppressor, were conducted at day/night temperatures with 26/24°C. To determine if P0-NY could function as a strong silencing suppressor at lower temperatures, either wild-type P0-NY or its mutant P0-NY(P-S) was co-expressed with a sGFP transgene in the *N*. *benthamiana* line 16c. Under these conditions, the VSR activity of wild-type P0-NY was similar to that of the strong suppressor P0-NY(P-S) at 6 dpi (S7C Fig).

Next, to analyze the protein accumulations of P0-NY or P0-NY(P-S) under different thermal stress conditions with autophagy inhibitor treatment, we used 3-MA to inhibit autophagy degradation. As shown in S8 Fig, at 20°C, both GFP-P0-NY and GFP-P0-NY(P-S) proteins were consistently detected and accumulated to the same level in leaves treated with 3-MA or DMSO (S8A Fig). However, at 28°C, GFP-P0-NY was barely detectable, and the protein accumulated to higher levels in leaves treated with 3-MA compared to those treated with DMSO (S8B Fig). These results suggest that P0-NY is less stable than P0-NY(P-S) under higher thermal stress and is subject to degradation through the autophagy pathway.

### The serine at locus 247 is required for P0-mediated viral pathogenicity under increasing thermal stress

**Fig 8:**
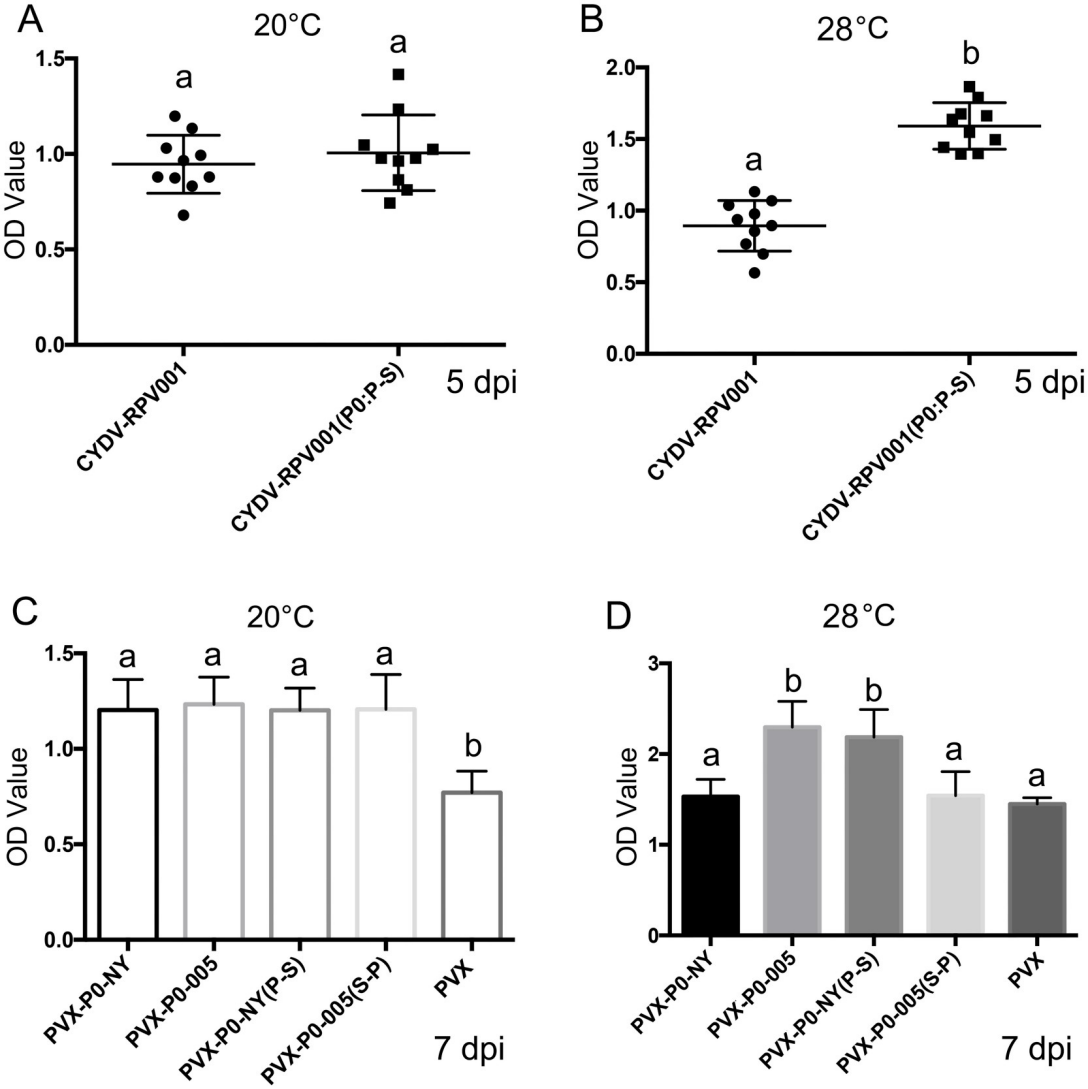
The C-terminal serine at amino acid 247 is required for P0-mediated viral pathogenicity. *N*. *benthamiana* leaves were infiltrated with *Agrobacterium* carrying either plasmid pCB-RPV001 or plasmid pCB-RPV001(P0:P-S), and ELISA was performed using the local infiltrated tissues incubated at either 20°C (A), or 28°C (B) at 5 dpi. Antigen was measured by DAS-ELISA in three randomly selected developing leaves from each of 10 infiltrated plants. *N*. *benthamiana* leaves were infiltrated with *Agrobacterium* carrying plasmid PVX-P0-NY, PVX-P0-005, PVX-P0-NY(P-S) or PVX-P0-005(S-P), and ELISA was performed using the systemically infected tissues at 7 dpi at either 20°C (C), or 28°C (D). PVX antigen was measured by DAS-ELISA in three randomly selected developing leaves from each of 15 plants. Letters (a, b) above the bars in (A), (B), (C), (D) indicate significant differences revealed by Dunn’s multiple comparisons test p<0.05.

To test the effect of P0 instability on viral pathogenicity, CYDV-RPV infectious clone pCB-RPV001 and pCB-RPV001(P0:P247S) was constructed and the latter contains only substitutions of nucleotide sequences encoding the proline at position 247 with those encoding serine. *Agrobacterium* carrying pCB-RPV001 or pCB-RPV001(P0:P247S) were infiltrated into *N*. *benthamiana* leaves and virus accumulation in the infiltrated leaves was quantified by DAS-ELISA at 5 dpi. Virus accumulation was similar between the two constructs at 20°C (Fig 8A), but at 28°C CYDV generated from pCB-RPV001(P0:P247S) accumulated to significantly higher titers than CYDV generated from pCB-RPV001at 5 dpi (Fig 8B).

To determine if temperature and P0 configuration influences CYDV systemic accumulation, *N*. *benthamiana* plants were infiltrated with *Agrobacterium* containing either pCB-CYDV-RPV001 or pCB-CYDV-RPV001(P0:P-S). Fifteen plants inoculated with each of these viruses were maintained at 20°C, 28°C or 30°C. At 4 wpi all but one of the plants maintained at 20°C was systemically infected (Table 1). Eleven of 15 plants inoculated with pCB-CYDV-RPV001(P0:P-S) (a strong silencing suppressor) and maintained at 28°C were systemically infected at 4 wpi, whereas four of 15 plants inoculated with pCB-CYDV-RPV001were systemically infected at this temperature. When plants were grown at 30°C, none of the 15 plants inoculated with pCB-CYDV-RPV001 developed a systemic infection, whereas eight of 15 plants infiltrated with pCB-CYDV-RPV001(P0:P-S) were infected systemically (Table 1). Taken together all the results suggest that CYDV-RPV isolates that select for a strong silencing suppressor will be maintained at higher temperatures and are likely to outcompete isolates that encode the weaker silencing suppressor, i.e., those containing a proline at position 247.

**Table 1:** Systemic infection efficiency of CYDV-RPV001 and CYDV-RPV001(P0:P-S) on *N*. *benthamiana* at three temperatures.

Virus source	Temperature		
	20°C	28°C	30°C
CYDV-RPV001	14/15^a^	4/15^b^	0/15^c^
CYDV-RPV001(P0:P-S)	15/15	11/15^b^	8/15^c^

^a^ Number of plants infected /Number of plants inoculated

^b, c^ Significant differences in the number of transmitted plant (P < 0.01 by one-way analysis of variance [ANOVA])

To further study the effects of temperature on P0 function, P0-NY and P0-NY(P-S) were expressed from *Potato virus X* (PVX). The *Agrobacterium* harboring PVX-P0-NY or PVX-P0-NY(P-S) was infiltrated into *N*. *benthamiana* plants at the 4-leaf stage, and the plants were maintained in growth chambers at 20°C or 28°C. By 3 wpi all the plants infected with PVX-P0-NY or PVX-P0-NY(P-S) and maintained at 20°C were dead (S9 Fig). The plants infected with PVX-P0-NY maintained at 28°C developed mild mosaic symptoms by 3 wpi, whereas the plants infected with PVX-P0-NY(P-S) all died (S9 Fig). DAS-ELISA was used to quantify PVX levels in systemically infected tissues at 7 dpi. When plants were maintained at 20°C, PVX co-infiltrated with any of the P0 constructs accumulated to significantly higher levels than when inoculated alone (Fig 8C), whereas at 28°C, only plants co-inoculated with PVX plus either P0-005 or P0-NY(P-S) accumulated increased levels of PVX (Fig 8D).

### The strong silencing suppressor conveys a selection advantage at higher temperatures

To test our competition hypothesis, we generated mixed infections of CYDV-RPV strains, containing either strong or weak silencing suppressors, in a natural plant host, oats. *Agrobacterium* containing either pCB-RPV001(P0-NY) or pCB-RPV001(P0:P-S) was infiltrated into *N*. *benthamiana* and virus was purified from infiltrated leaves. Equal amounts of purified CYDV-RPV001 and CYDV-RPV001(P0:P-S) were mixed in 15% sucrose and fed to aphids (*R*. *padi*) using Parafilm sachets, and oat plants were infested with the viruliferous aphids. Plants were incubated under day/night temperature regimes of either 20/16 ±1°C or 28/24 ±1°C, and the infection status of the plants was determined by DAS-ELISA at 4 wpi. The ELISA positive plants were further tested using RT-PCR and sequencing to determine the proportion of P0 genes encoding proline or serine at position 247. Amplicons spanning CYDV-RPV genome position 382 to 1081, which includes the nucleotides encoding P0 amino acid 247, were cloned into the pJET1.2 vector. The P0 sequences were determined in plasmids of three colonies containing RT-PCR products obtained from each infected plant. Twelve of the 19 infected plants maintained at the lower temperatures were found to contain a mixture of both viruses; 17 of the plasmids contained a sequence encoding a proline at P0 position 247 and 19 of the plasmid clones contained a sequence encoding a serine at P0 position 247 (Table 2). Three of the 19 plants were infected with CYDV-RPV001(P0:P-S) alone and four of the 19 plants were infected with CYDV-RPV001 alone. In contrast, only two of 15 infected plants maintained at the higher temperatures were found to contain both viruses; two of the six clones analyzed contained sequences encoding a proline, while four of the six clones analyzed contained sequences encoding a serine. The remaining 13 plants were infected with CYDV-RPV001(P0:P-S) alone (Table 2). The results above indicated that an S247 CYDV-RPV provided a selective advantage at the higher temperature and could play a significant role in virus competition in warming climates.

**Table 2:** Aphid transmission of mixture of CYDV-RPV001 and CYDV-RPV001(P0:P-S) on oat plants at two temperatures, and characterization of P0 in the infectious progeny virus.

Virus	Temperature (Day/Night)	
	20/16 ±1°C	28/24 ±1°C
Aphid transmissibility^a^	19/30	15/30
Mixed infection^b^	12/19 (17:19)^c^	2/15 (2:4)^c^
CYDV-RPV001 single infection^d^	4/19^e^	0/15^e^
CYDV-RPV001(P0:P-S) single infection^d^	3/19^f^	13/15^f^

^**a**^ Aphid transmissibility (Number plants infected /Number plants inoculated)

^b^ Number of mixed infected plants / Total number of infected plants

^**c**^ The number of plasmid clones containing sequences encoding proline vs. the number of plasmid clones containing sequences encoding serine in P0, from three plasmids sequenced / RT-PCR product derived from viral RNA extracted from each infected plant

^d^ Number of plants singly infected with this variant of P0 / Total number of infected plants

^e, f^ Significant differences in the number of infected plants (P < 0.05 by one-way analysis of variance [ANOVA])

## Discussion

P0 is one of the most variable ORFs in poleroviruses and contains the highest frequency of sites under positive selection [35,36]. The intra-species diversity of polerovirus P0s silencing suppression has been shown to be high. In this study, by comparing the sequences of P0 from two different CYDV-RPV isolates, we demonstrated that a single C-terminal amino acid in the CYDV-RPV P0 could influence P0 suppressor activity by influencing protein stability. This finding is exhilarating considering this naturally occurring substitution is not in the F-box like motif, GW motif or C-terminal aromatic acid region which all these motifs are vital for P0 suppressor activity. The polerovirus P0 minimal F-box motif interacts with the SCF family of E3-ligase SKP1 components and stimulates AGO degradation through the autophagy pathway [14,25]. The BYV P0 interacts with host SKP1 to prevent virus degradation by the proteasome and autophagy pathways [18]. Furthermore, the interactions of polerovirus P0 proteins with ASK proteins were observed to be temperature restricted, e.g., the interaction of TuYV P0 with ASK1 or ASK2 was observed at 21°C, but not 28°C [25]. Similarly, *Beet western yellows virus* P0 interacts only with ASK1 and ASK2 at 21°C other than at 28°C in the yeast two-hybrid assay and the *Cucurbit aphid-borne yellows virus* P0 interactions with ASK1 and ASK2 were stronger at 21°C than 28°C [25,37]. But the reasons for the effect of temperature on the interaction between the P0s and the ASK proteins is not well documented. Here we showed that silencing suppressor activity could be strengthened by mutation of the single C-terminal proximal proline in CYDV-RPV NY, an isolate that has weak suppressor activity, to serine found in CYDV-RPV isolates with a strong suppressor activity. This change did not alter the interaction of P0 with SKP1 proteins from either natural host barley or *N*. *benthamiana*. While this result was not surprising since the F-box motif all CYDV-RPV P0s is conserved, and we showed strong evidence that the weak CYDV-RPV P0 VSR containing the proline residue was unstable at high temperatures, both in yeast and *in planta*.

Protein stability often determines viral fitness and is one of the major drivers of protein evolution [38]. Many mutational effects on fitness parameters have been associated with changes in the thermodynamic stability of viral proteins [39,40]. For example, a Y161F hemagglutinin substitution increases thermostability and improves yields of H1N1 influenza A virus in cells [41]. Effects of a residue in influenza A virus involved in viral replication and protein expression levels were likely attributable to structural stability rather than functional constraints [42]. A single amino acid substitution in the capsid of foot-and-mouth disease virus increased acid resistance [43]. Proline is the only cyclic amino acid, and it often plays unique roles in protein folding, structure stabilization, and protein interactions. While the unique cyclic structure of its side chain provides exceptional conformational rigidity relative to other amino acids, its presence is often associated with a destabilization of protein structure [44,45]. Substitution of proline residues is linked to changes in protein function [46–49], and proline-to-alanine substitutions can significantly increase thermodynamic stability of onconase protein [50]. Here we report that substitution of proline with other amino acids (alanine, aspartic acid, and serine) increased P0 stability, suggesting that the exceptional conformational rigidity of proline somehow destabilizes the P0 protein structure. Furthermore, the destabilization was found to be temperature dependent. Structural analysis of the P0 in the future is needed to clarify the roles of P247 in the CYDV-RPV P0.

Temperature is an environmental parameter that differentially affects the interaction of compatible hosts with different RNA viruses [51,52]. Moreover, temperature-sensitive mutations in viruses are long recognized as a type of mutations that occur in many different genes controlling a variety of different functions. They have been found in almost all viruses including bacteriophages, plant viruses and animal viruses. For example, the *Tobacco mosaic virus* temperature-sensitive mutant Lsl [53–55], located in the gene encoding the 30K movement protein [56,57] prevents the virus from moving into adjacent healthy cells at the restrictive temperature (33°C) [55]. Accelerating climate warming and extreme weather events are becoming more frequent and pose an increased risk for crops to become infected with pathogens, which could severely affect global food security [58]. There are studies indicating that global climate change is driving worldwide genetic changes in mammalian viruses, prokaryote and eukaryotic organisms [59,60]. But whether climate change could drive genetic changes of plant viruses in ecological level is unknown.

In the present study, we demonstrated that CYDV-RPV isolates with a VSR containing a serine residue at position 247, were able to infect host plants at higher temperatures and accumulated to higher titers in those plants, allowing the plants to serve as more efficient virus reservoirs and more abundant sources of virus for aphids in warming climates. Because CYDV-PRV containing S247 in its P0 protein outcompetes CYDV-PRV containing P247 in its P0 at higher temperatures, the S247 allele may be more prevalent than P247 variant in warmer environments. Noteworthily, all isolates collected after the year of 2007 was found with serine at loci 247 of P0. Thus, as the global climate warms, we predict that viruses with the S247 P0 genotype will become more abundant relative to those containing P247. A question remains as to whether the P247 genotype, with a shorter P0 half-life, may confer a selective advantage in certain environmental conditions, or, because it has been found in only few isolates, it may be a rare, non-beneficial mutation. Regardless of that possibility. this research provides a potential mechanistic explanation for how climate change could affect host-virus interactions at the molecular level.

## Materials and methods

### Plasmid constructions

pCB-RPV001, which contains the CYDV-RPV genome under the control of the *Cauliflower mosaic virus* 35S promoter and terminated by a ribozyme and nos termination signal sequences [22], was constructed by subcloning pCNYfull51 that contains the full-length, infectious genome of CYDV-RPV (New York isolate) fused to a bacteriophage T7 gene promoter. pCNYfull51 was prepared by full-length, end-to-end RT-PCR using primers matched to each end of the CYDV-RPV genomic RNA (accession no. NC_004751).

The P0 of CYDV-RPV New York isolate (P0-NY, GenBank No. NC_004751) was cloned from pCB-RPV001 into a binary vector with a C-terminal flag tag (pBlin-P0-NY-flag). P0 of CYDV-RPV 005 isolate (GenBank No. EF521827.1, P0-005) was synthesized and cloned using P0-NY as the template following the manufacturer’s instruction (GeneArt Site-Directed Mutagenesis Kit).

Constructs pBin-35S-mGFP, pBin:P19 (*Cymbidium ringspot virus*) and 35S:hpGFP were described previously [61]. The P0 cDNA of CYDV-RPV-NY (NC_004751) was amplified by PCR from the full-length cDNA clone pCB-RPV001. The P0 of CYDV-RPV-005 (EF521827.1) was synthesized and ligated into the pJET1.2 vector using the GeneArt Site-Directed Mutagenesis System. P0-NY (P-S) and P0-005 (S-P) were generated by overlap PCR. After digestion using the restriction enzymes *Kpn*I and *Bam*HI, the P0-containing fragments were ligated individually to either the vector pBlin such that the encoded C-terminus of P0 was fused to a flag tag (pBlin-P0-flag) or the vector pBlin-GFP, such that the encoded N-terminus of P0 was fused to the sequences encoding the C-terminus of a GFP tag (pBlin-GFP-P0). These fragments were also ligated individually to the vector pGBKT7 vector for the yeast two-hybrid assay.

To construct the PVX P0 expression vectors, wide-type P0-NY, P0-005, and mutants P0-NY (P-S) and P0-005(S-P) were PCR amplified with primers containing *Cla*I and *Sal*I restriction sites. The PCR fragments were ligated individually into the pJET1.2 vector (Thermo Fisher, Waltham, USA) and were transformed into *E*. *coli* DH5α. After isolation of the plasmids from the resulting colonies, they were digested using the enzymes *Cla*I and *Sal*I, and the resulting inserts were ligated individually into the PVX (pgR106) vector as described elsewhere [62]. All the plasmids were verified by DNA sequencing before further use. Leaves of 4-week-old plants were infiltrated with *A*. *tumefaciens* (strain GV3101) harboring these PVX plasmids using needleless syringes as described previously [62].

The NbSKP1 from *N*. *benthamiana* and HvSKP1 from barley were amplified by RT-PCR from the corresponding cDNAs with the primers NbSKP1_F (5′- GAATTCATGAAGATGATCGTGCTAAGGAGTTC-3′) / NbSKP1_R (5′-GGATCCTTACTCGAAGGCCCAGGCATTCTC-3′) and HvSKP1_F (5′- GAATTCATGGCGGCCGCTGGAGAC-3′) / HvSKP1_R (5′-GGATCCCTACTCAAAGGCCCACTGGTTCTC-3′). The PCR products were then ligated into the vector pJET1.2 (Thermo fisher, USA) and transformed into Top10. After digestion of the isolated plasmid, using the enzymes *Ecol*RI and *Bam*HI, the fragments containing the SKP1 gene were ligated individually to the vector PGADT7. The construct integrity was confirmed by sequencing.

### Plant growth conditions

All the *N*. *benthamiana* plants used in this assay were grown in a growth chamber set at at day/night temperatures with 26/24°C and 16h/8h light/dark conditions, except those used in temperature-shift experiment. The oat plants were grown and maintained at 18 ± 1°C, 16:8 (L:D), except those used in temperature-shift experiment.

### RNA silencing suppression activity in *N*. *benthamiana* and in transgenic *GFP N*. *benthamiana* 16c plants

All constructs used for agroinfiltration were electroporated into *A*. *tumefaciens* strain C58C1 with a Gene Pulser II system (Bio-Rad). For co-infiltration assays, *A*. *tumefaciens* containing various plasmids were grown individually to an OD_600_ of 0.6–0.8. The cultures were pelleted and resuspended in an infiltration medium containing 20 mM MgCl_2_, 10 mM MES (pH 5.6), and 100 μM acetosyringone to give a final OD_600_ of 1.0. Equal volumes of *A*. *tumefaciens* cultures harboring either the plasmids with VSRs, 35S-GFP, and 35S-dsGFP, or the plasmids with VSRs and 35S-GFP were mixed and infiltrated into leaf tissues of either 4-week-old *N*. *benthamiana* or transgenic *GFP N*. *benthamiana* 16c plants, respectively, by using 1-ml syringes without needles. The leaves were photographed under UV illumination (with a UV Products lamp) at different dpi using a digital camera.

### Confocal microscopy observation and GFP fluorescence quantification

To visualize the subcellular localization of P0 variants, leaves of 4-5-week *N*. *bethamiana* plants were infiltrated with *A*. *tumefaciens* (strain GV3101) harboring GFP-P0-NY and its variant plasmids. Agrobacterium suspensions at 0.8 optical density (OD_600_) was used for infiltration. Leaf tissue was harvested at 48h or 72h post agroinfiltration and examined for GFP fluorescence using a Leica TCS-SP5 confocal microscope (Leica Microsystems).

For GFP quantification, total proteins were extracted from six leaf discs, taken from the infiltrated leaf area, with an extraction buffer (100 mM KCl, 5 mM MgCl2, 400 mM sucrose, 100 mM Tris–HCl pH 8, 10% glycerol, 2 mM PMSF, EDTA-free protease inhibitor cocktail (Sigma). Total protein concentration was determined using a protein assay kit (Bio-Rad). GFP detection was performed by western blot with 10 μg total proteins.

For BiFC assay, P0-NY, P0-NY(P-S), NbSKP1, and HvSKP1 were fused to the C-terminal or N-terminal fragment of YFP in the Yn and Yc vectors, respectively, and then were used for agroinfiltration into *N*. *benthamiana*. At 2 dpi, the interactions were observed for GFP fluorescence using a Leica TCS-SP5 confocal microscope (Leica Microsystems).

### Yeast two-hybrid assay

Experiments were performed as described by Xu *et al* [63]. CYDV P0-NY and P0-NY (P247S) were cloned into pGBKT7. NbSKP1 and HvSKP1 were individually cloned into the pGADT7 vector. Double transformants were streak-replicated on either SD medium lacking histidine, tryptophan and leucine (-HWL), or, for more stringent selection, on SD medium lacking adenine, histidine, tryptophan and leucine (-Ade, -His, -Trp, -Leu). Plates were incubated at 22°C and 30°C, respectively.

### Chemical treatments, in vivo protein degradation assay

In vivo protein degradation analysis was performed as described previously [64] with minor modification. For in vivo analysis of P0 degradation, *A*. *tumerfaciens* strain C58C1 harboring GFP-P0-NY or GFP P0-NY(P-S) constructs were infiltrated into *N*. *benthamiana* leaves. Sixteen hours before sampling, the indicated concentrations of CHX (100 μM, Sigma, USA), the inhibitor 3-MA (5 mM, Aladdin, China), E64d (50 μM, Sigma, USA) or an equal volume of DMSO control solution, were infiltrated. Confocal microscopy was used to observe the GFP fluorescence at 3 dpi. For western blot analysis, protein from each of the infiltrated tissues was extracted at 3 dpi and then P0(s) accumulation was confirmed.

### Virus induced gene silencing

*Agrobacterium* was prepared as above carrying TRV1 and the appropriate TRV2 construct and mixed to a final OD_600_ of 0.4, in agroinfiltration buffer supplemented with 100 μM acetosyringone (Sigma) and left in the dark for 2–3 h prior to infiltration. Four-to-five leaves of *N*. *benthamiana* seedlings were infiltrated in both cotyledons and the leaves that had emerged. *N*. *benthamiana* plants were infiltrated with TRV1 and TRV2-ATG3 for ATG3-silencing, TRV1 and TRV2-ATG7 for ATG7-silencing and TRV1 and TRV2-EV for the empty vector control. TRV2 containing the *N*. *benthamiana phytoene desaturase* (PDS) gene fragment (TRV2-NbPDS) was used as a positive control to indicate viral spread. Plants were left to grow under standard conditions until the effects-of-gene-silencing experiments could be carried out, three weeks later.

### Aphid transmission assays

*Agrobacterium* containing either pCB-RPV001 or pCB-RPV001(P0:P-S) was infiltrated into *N*. *benthamiana* and virus was purified from infiltrated leaves. Equal amounts of CYDV-RPV001 and CYDV-RPV001(P0:P-S) were mixed in 15% sucrose and fed to aphids (*R*. *padi*) using Parafilm sachets, and oat plants were infested by the viruliferous aphids. Adult aphids were allowed a 24 h acquisition access period on the parafilm sachets and five aphids were transferred to each of 30 oat plants and allowed a five-day inoculation access period (18 ± 1°C, 16:8 L:D), prior to being fumigated. Plants were moved to growth chambers maintained at either day/night temperatures of 20/16 ±1°C or day-night temperatures of 28/24 ±1°C. Plants were incubated under day/night temperature regimes of either 20/16 ±1°C or 28/24 ±1°C, and the infection status of the plants was determined by DAS-ELISA at 4 wpi.

## Supporting information

S1 Fig Alignment of the C-terminus amino acids of CYDV-RPV P0 in all CYDV sequences available in GenBank. Each logo consists of stacks of symbols, one stack for each position in the sequence. The overall height of the stack indicates the sequence conservation at that position, while the height of symbols within the stack indicates the relative frequency of each amino at that position. The red arrows indicate the position of the amino acid 247.
(TIF)

S2 Fig Differences in P0 protein accumulation in leaves transiently expressing P0-NY, P0-005 and P0 amino acid 247 substitution mutants with the addition of cycloheximide. (A) Leaves of the *N*. *benthamiana* plants were agroinfiltrated with bacteria containing pBlin-P0-NY-flag, pBlin-P0-005(S-P)-flag, pBlin-P0-005-flag or pBlin-P0-NY(P-S)-flag. Sixteen hours before sampling, the indicated concentrations of CHX or equal volume of DMSO control solution were infiltrated. Protein from each of the infiltrated tissues was extracted at 3 dpi and 4 dpi and then P0-flag accumulation was confirmed by western blot analysis.
(TIF)

S3 Fig Localization and differences in P0 protein accumulation in leaves transiently expressing P0-NY, P0-005 and P0 amino acid 247 substitution mutants. (A) Leaves of the *N*. *benthamiana* plants were agroinfiltrated with bacteria containing pBlin-GFP-P0-NY, pBlin-GFP-P0-005(S-P), pBlin-GFP-P0-005, pBlin-GFP-P0-NY(P-S), pBlin-GFP-P0-Rochow or pBlin-GFP-P0-Rochow(S-P). The GFP-P0 accumulation was observed under confocal microscopy using 150 V at 2 dpi and 3 dpi, respectively. (B) GFP-P0-NY frequently concentrated to the inclusion bodies near the perinuclear area, like aggresomes. Arrows represent the inclusion bodies near the nuclear.
(TIF)

S4 Fig Structure and phosphorylation prediction of P0-005. (A) Structure-based method for predicting the fold stability change after P0 247 changes from serine to proline; ΔΔG below zero means the mutation causes destabilization. Serine was labeled in pink. (B) The potential phosphorylation site in P0-005 was predicted by using the Group-based prediction system (http://gps.biocuckoo.cn).
(TIF)

S5 Fig The degradation of P0 (s) is compromised when the autophagy pathway is impaired. In vivo degradation inhibition analysis of P0-NY and P0-NY(P-S) accumulation after adding autophagy pathway inhibitors 3-MA or E64d (A). (B) Effect of NbATG3, NbATG7 knockdown on the protein stabilization of GFP-P0-NY in *N*. *benthamiana*. Bars: 50 μm.
(TIF)

S6 Fig Detection of interaction interactions of P0-NY and P0-NY (P-S) with NbSKP1 and HvSKP1 using bimolecular fluorescence complementation (BiFC) assay. The fluorescence was detected by confocal microscope at 2 dpi. Bars: 100 μm.
(TIF)

S7 Fig P0 shows instability to increasing thermal stress and P0-NY can suppress gene silencing at 20°C. *N*. *benthamiana* plants were infiltrated with *Agrobacterium* harboring plasmid pBin-GFP-P0-NY (a, c, e), or plasmid pBin-GFP-P0-NY(P-S) (b, d, f). a, b: plants were kept at 20°C; c, d: plants were kept at 24°C; e, f: plants were kept at 28°C. The GFP-P0 accumulation was monitored by confocal microscopy using 150 V at 2 dpi. (B) GFP-P0 expression was measured by western blot in different temperature conditions at 2 dpi. The relative protein level of P0-NY at 20°C was normalized to rubisco, and this value was set as standard 100. (C) Leaves of the *N*. *benthamiana* 16c plants were co-agroinfiltrated with a GFP-expressing vector (GFP) and either pBlin-P0-NY or pBlin-P0-NY(P-S). The plants were kept at a chamber setting of 20°C. The leaves were photographed at 6 dpi under a hand-held long-wavelength UV lamp. The expression of GFP and P0-flag were detected by western blot using GFP antibody and flag antibody, respectively.
(TIF)

S8 Fig The protein accumulations of P0-NY or P0-NY(P-S) with autophagy inhibitor treatment under different thermal stress conditions. In vivo degradation inhibition analysis of P0-NY and P0-NY(P-S) accumulation after adding autophagy pathway inhibitors 3-MA at 2 dpi under different thermal stress conditions 20°C (A) and 28°C (B). *A*. *tumefaciens* containing pBlin-GFP-P0-NY and pBlin-GFP-P0-NY(P-S) constructs were infiltrated for transient expression side by side on either halves of independent *N*. *benthamiana* leaves, 16 h prior to inhibitor treatment. DMSO infiltration was used as a control. The GFP-P0 accumulation was monitored by western blot. Bars: 50 μm.
(TIF)

S9 Fig P0-NY and P0-NY(P-S) expressed in the heterologous *Potato virus X* system. Top panel: plants infected with PVX-P0-NY or PVX-P0-NY(P-S) all died when maintained at 20°C. Bottom panel: plants infected with PVX-P0-NY and maintained at 28°C developed mild mosaic symptoms by 3 wpi, whereas the plants infected with PVX-P0-NY(P-S) all died.
(TIF)

S1 Movie The trafficking of inclusion bodies near the perinuclear space. **GFP-P0-NY was infiltrated into *N*. *benthamiana* leaves**. The GFP fluorescence was visualized by confocal microscopy at 2 dpi.
(MOV)
