# Supplementary figures and images for: Naturally occurring substitution of an amino acid in a plant virus gene-silencing suppressor enhances viral adaptation to increasing thermal stress
Source: PLoS Pathog. 2023 Apr 3;19(4):e1011301. doi: 10.1371/journal.ppat.1011301 (PMC10101640; doi:10.1371/journal.ppat.1011301)

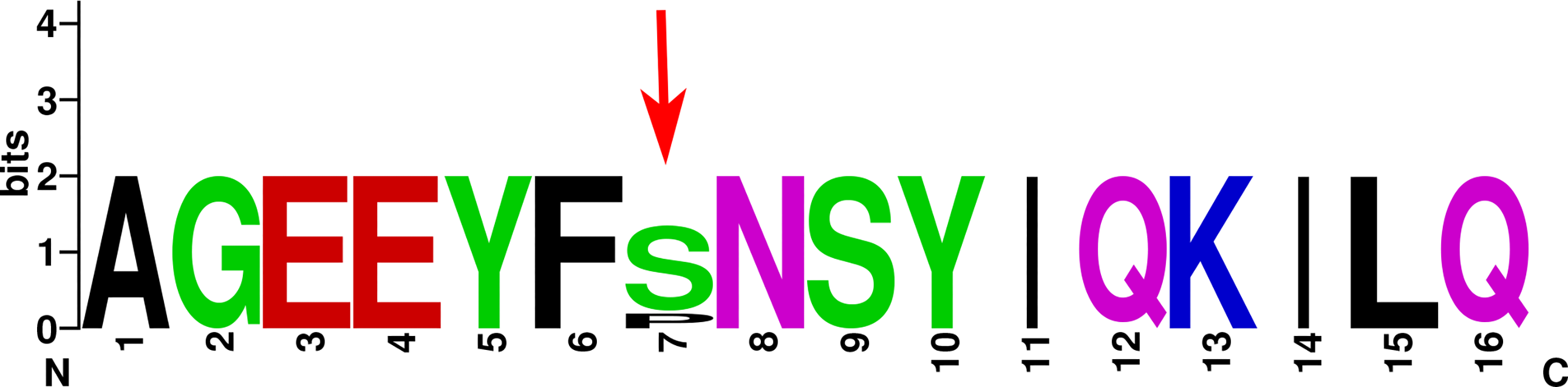

Supplement: S1 Fig — Each logo consists of stacks of symbols, one stack for each position in the sequence. The overall height of the stack indicates the sequence conservation at that position, while the height of symbols within the stack indicates the relative frequency of each amino at that position. The red arrows indicate the position of the amino acid 247. (TIF) [file ppat.1011301.s001.tif]

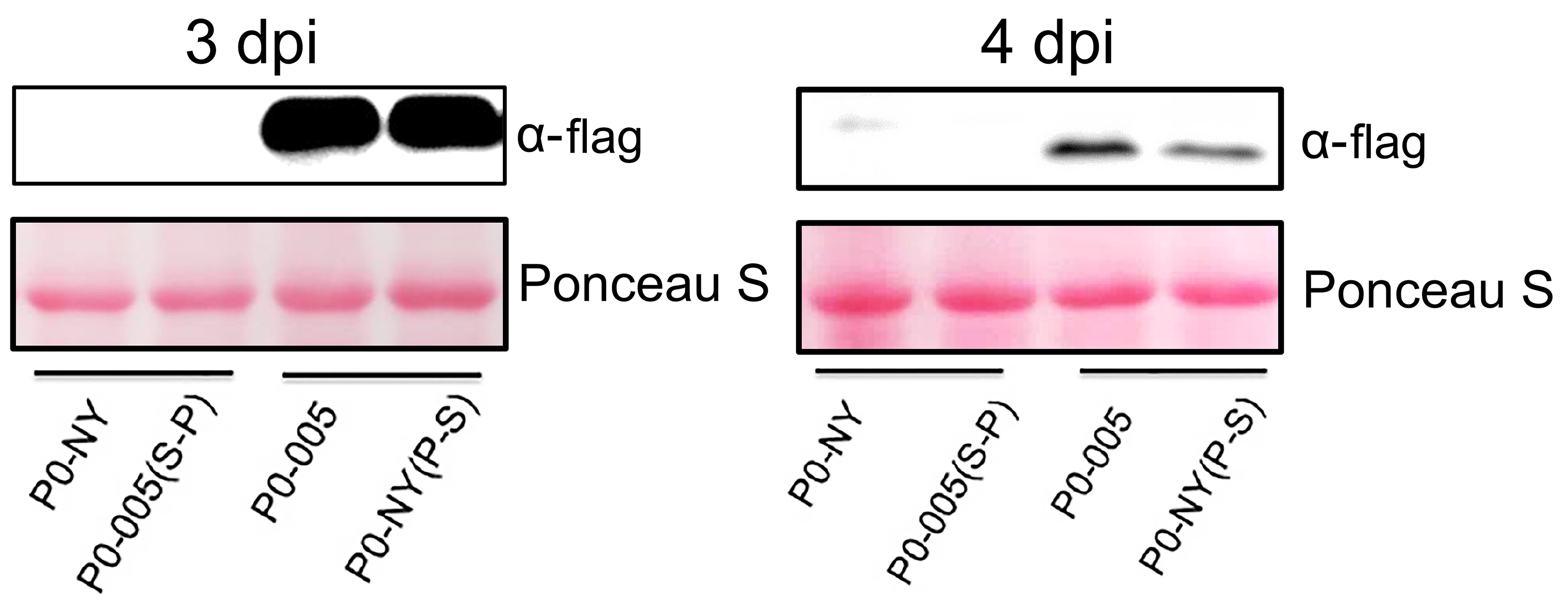

Supplement: S2 Fig — (A) Leaves of the N. benthamiana plants were agroinfiltrated with bacteria containing pBlin-P0-NY-flag, pBlin-P0-005(S-P)-flag, pBlin-P0-005-flag or pBlin-P0-NY(P-S)-flag. Sixteen hours before sampling, the indicated concentrations of CHX or equal volume of DMSO control solution were infiltrated. Protein from each of the infiltrated tissues was extracted at 3 dpi and 4 dpi and then P0-flag accumulation was confirmed by western blot analysis. (TIF) [file ppat.1011301.s002.tif]

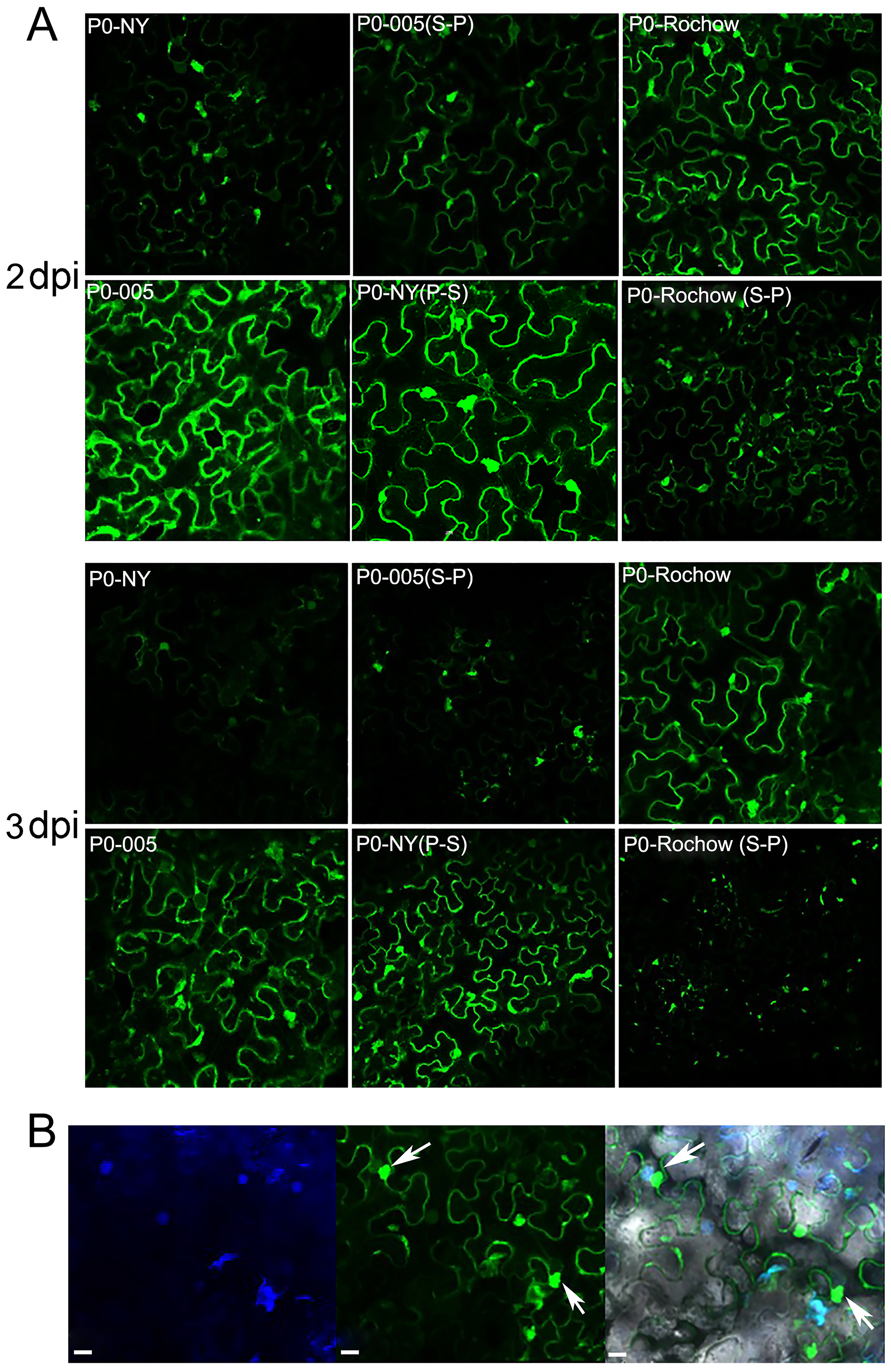

Supplement: S3 Fig — (A) Leaves of the N. benthamiana plants were agroinfiltrated with bacteria containing pBlin-GFP-P0-NY, pBlin-GFP-P0-005(S-P), pBlin-GFP-P0-005, pBlin-GFP-P0-NY(P-S), pBlin-GFP-P0-Rochow or pBlin-GFP-P0-Rochow(S-P). The GFP-P0 accumulation was observed under confocal microscopy using 150 V at 2 dpi and 3 dpi, respectively. (B) GFP-P0-NY frequently concentrated to the inclusion bodies near the perinuclear area, like aggresomes. Arrows represent the inclusion bodies near the nuclear. (TIF) [file ppat.1011301.s003.tif]

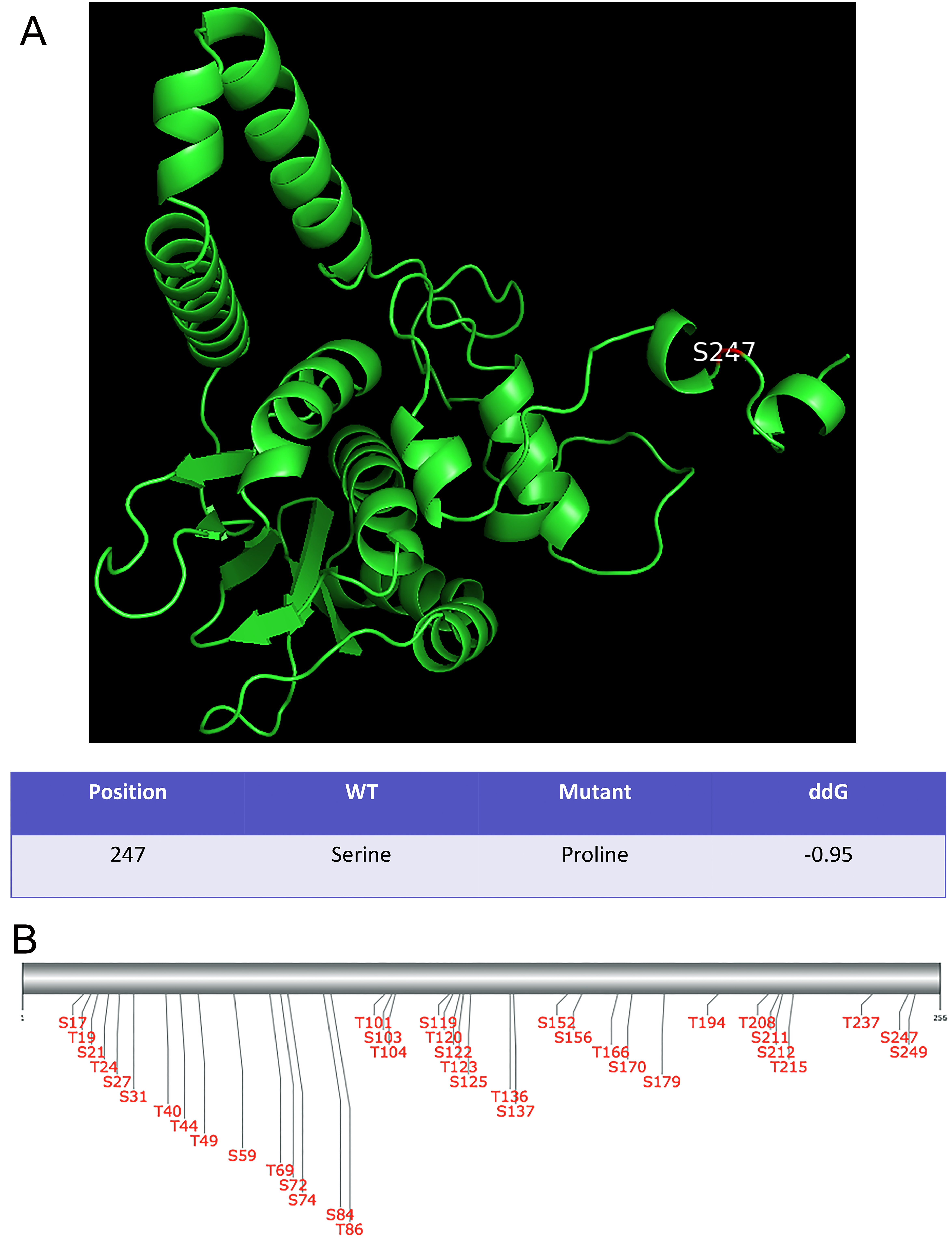

Supplement: S4 Fig — (A) Structure-based method for predicting the fold stability change after P0 247 changes from serine to proline; ΔΔG below zero means the mutation causes destabilization. Serine was labeled in pink. (B) The potential phosphorylation site in P0-005 was predicted by using the Group-based prediction system (http://gps.biocuckoo.cn). (TIF) [file ppat.1011301.s004.tif]

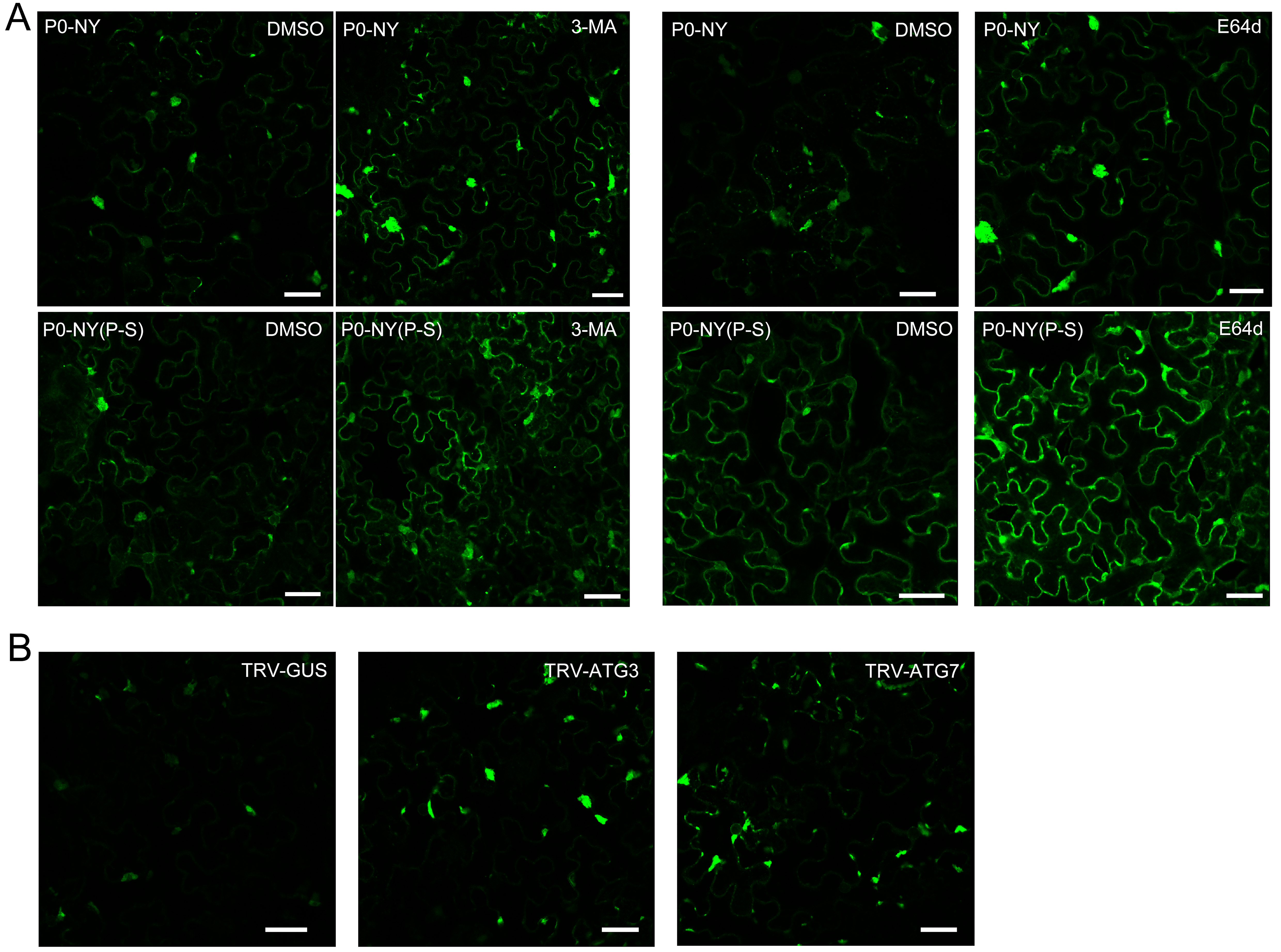

Supplement: S5 Fig — In vivo degradation inhibition analysis of P0-NY and P0-NY(P-S) accumulation after adding autophagy pathway inhibitors 3-MA or E64d (A). (B) Effect of NbATG3, NbATG7 knockdown on the protein stabilization of GFP-P0-NY in N. benthamiana. Bars: 50 μm. (TIF) [file ppat.1011301.s005.tif]

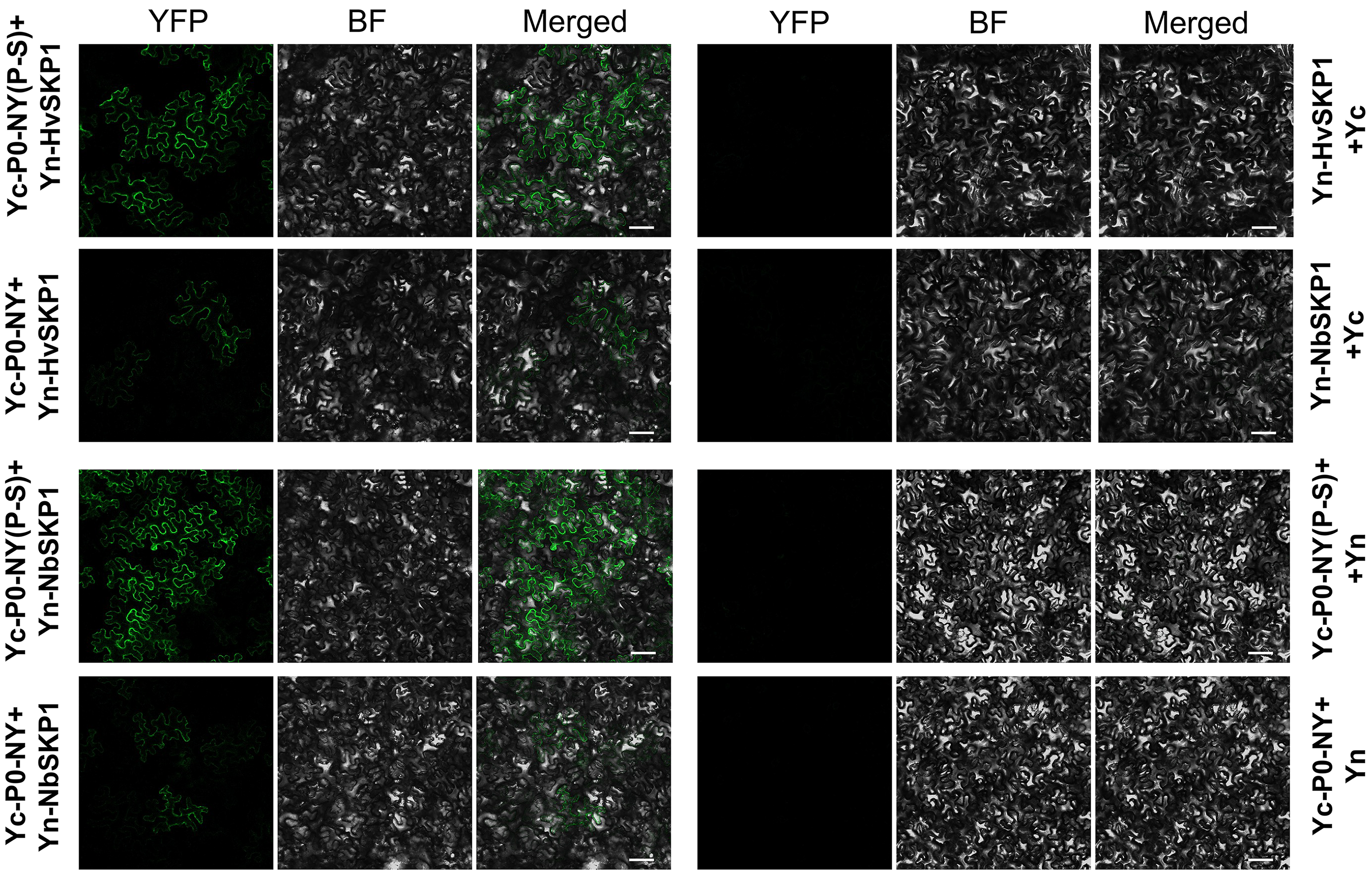

Supplement: S6 Fig — The fluorescence was detected by confocal microscope at 2 dpi. Bars: 100 μm. (TIF) [file ppat.1011301.s006.tif]

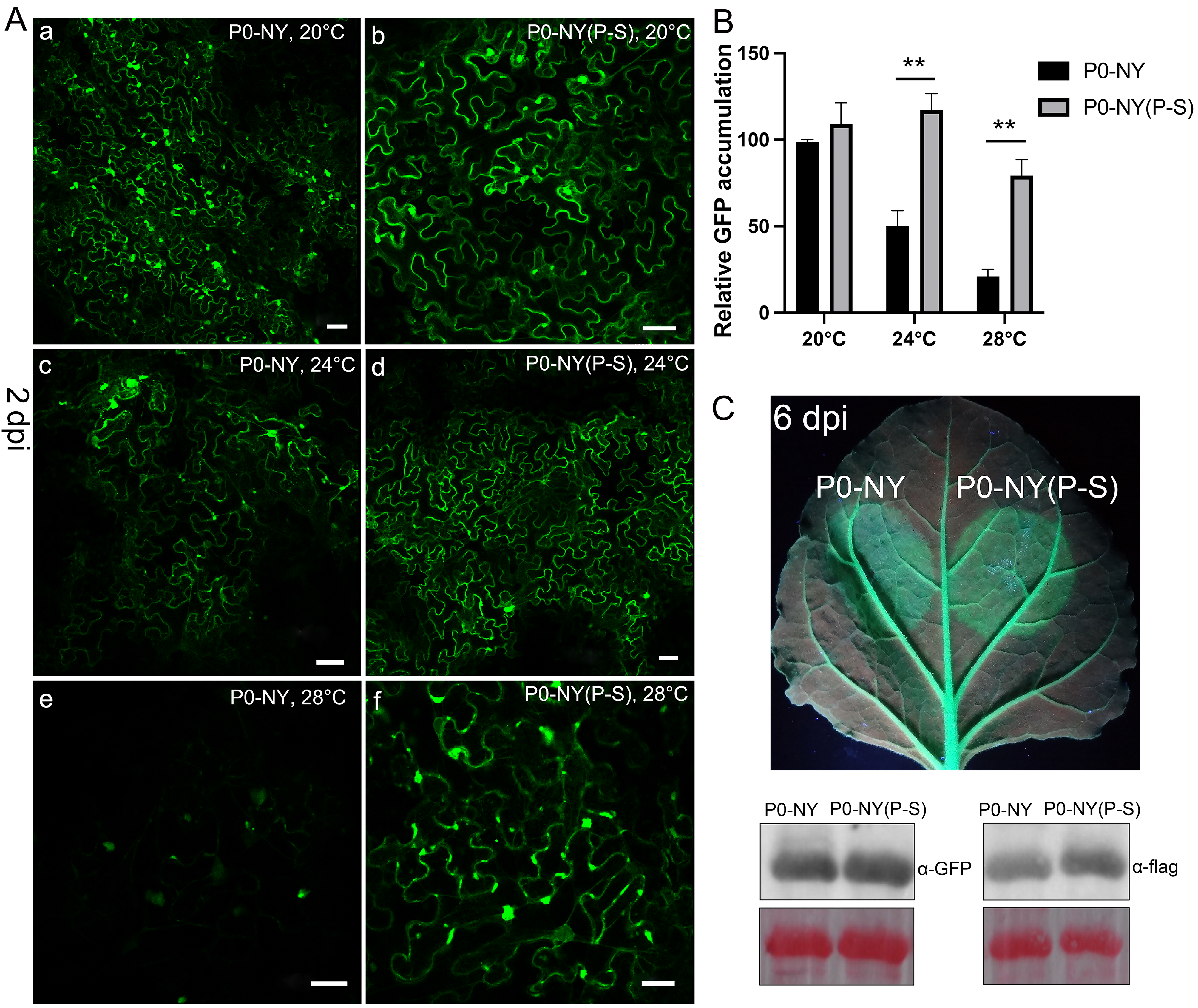

Supplement: S7 Fig — N. benthamiana plants were infiltrated with Agrobacterium harboring plasmid pBin-GFP-P0-NY (a, c, e), or plasmid pBin-GFP-P0-NY(P-S) (b, d, f). a, b: plants were kept at 20°C; c, d: plants were kept at 24°C; e, f: plants were kept at 28°C. The GFP-P0 accumulation was monitored by confocal microscopy using 150 V at 2 dpi. (B) GFP-P0 expression was measured by western blot in different temperature conditions at 2 dpi. The relative protein level of P0-NY at 20°C was normalized to rubisco, and this value was set as standard 100. (C) Leaves of the N. benthamiana 16c plants were co-agroinfiltrated with a GFP-expressing vector (GFP) and either pBlin-P0-NY or pBlin-P0-NY(P-S). The plants were kept at a chamber setting of 20°C. The leaves were photographed at 6 dpi under a hand-held long-wavelength UV lamp. The expression of GFP and P0-flag were detected by western blot using GFP antibody and flag antibody, respectively. (TIF) [file ppat.1011301.s007.tif]

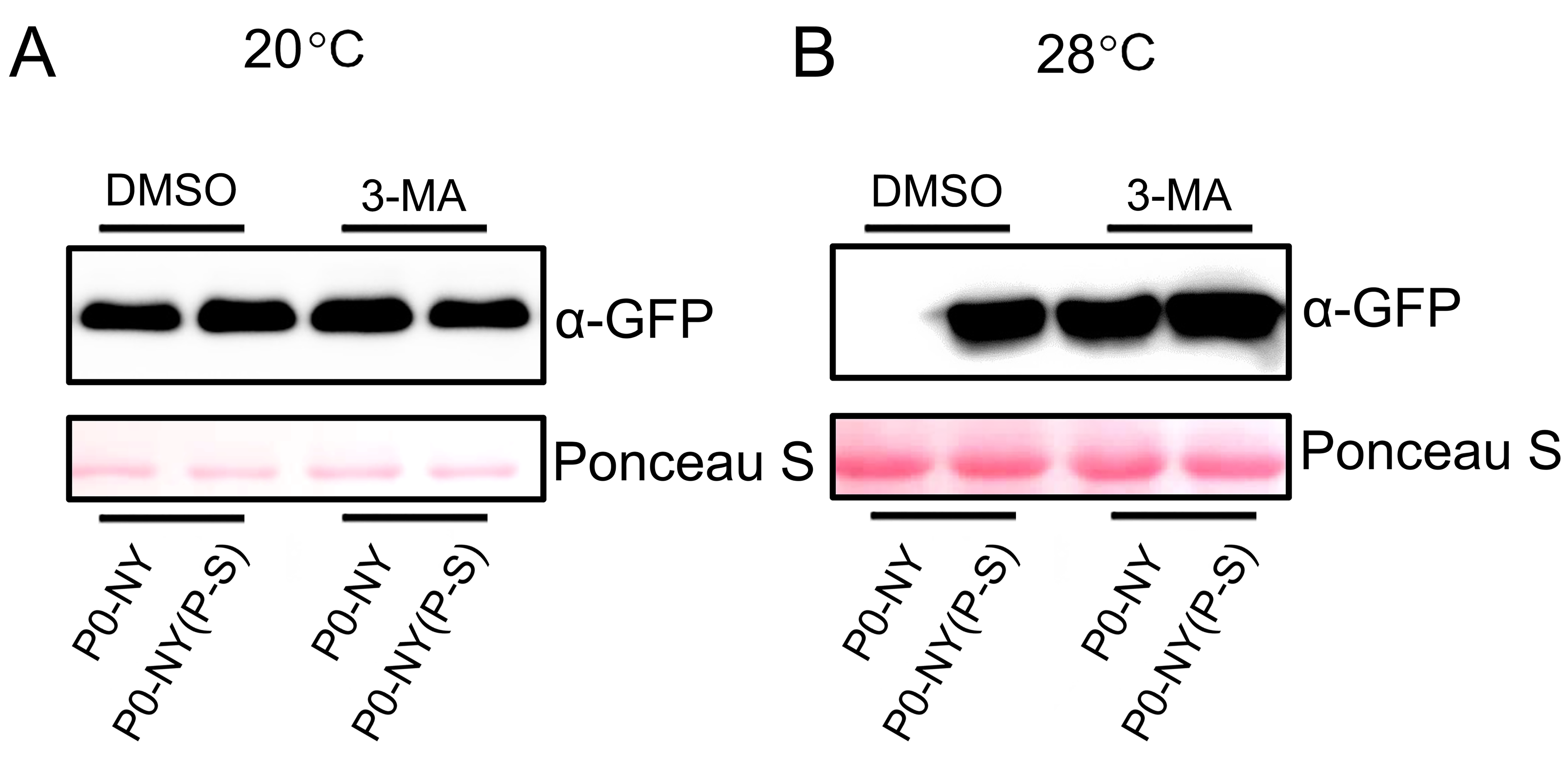

Supplement: S8 Fig — In vivo degradation inhibition analysis of P0-NY and P0-NY(P-S) accumulation after adding autophagy pathway inhibitors 3-MA at 2 dpi under different thermal stress conditions 20°C (A) and 28°C (B). A. tumefaciens containing pBlin-GFP-P0-NY and pBlin-GFP-P0-NY(P-S) constructs were infiltrated for transient expression side by side on either halves of independent N. benthamiana leaves, 16 h prior to inhibitor treatment. DMSO infiltration was used as a control. The GFP-P0 accumulation was monitored by western blot. Bars: 50 μm. (TIF) [file ppat.1011301.s008.tif]

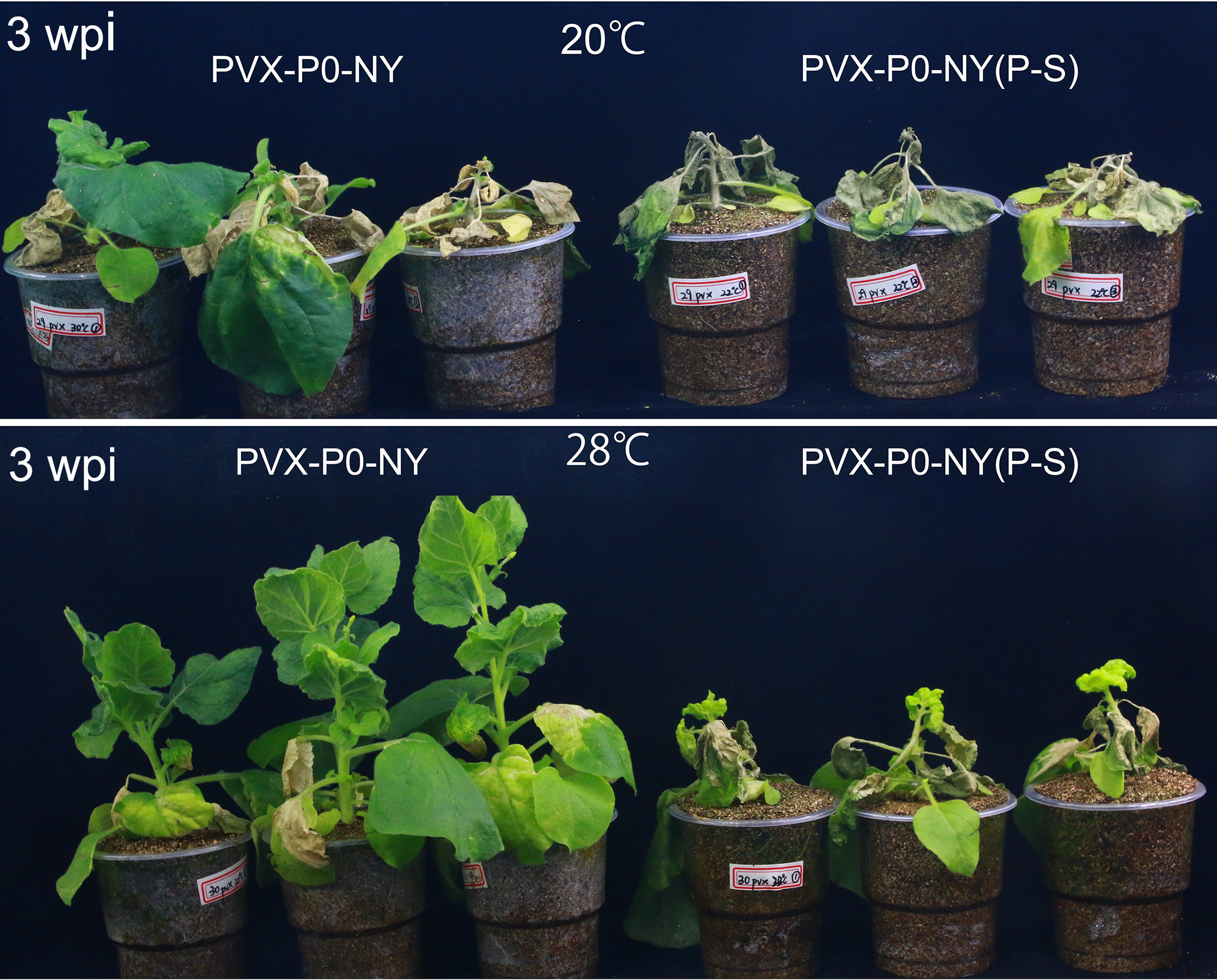

Supplement: S9 Fig — Top panel: plants infected with PVX-P0-NY or PVX-P0-NY(P-S) all died when maintained at 20°C. Bottom panel: plants infected with PVX-P0-NY and maintained at 28°C developed mild mosaic symptoms by 3 wpi, whereas the plants infected with PVX-P0-NY(P-S) all died. (TIF) [file ppat.1011301.s009.tif]
